# Biological characteristics of endometriotic mesenchymal stem cells isolated from ectopic lesions of patients with endometriosis

**DOI:** 10.1186/s13287-020-01856-8

**Published:** 2020-08-08

**Authors:** Yanli Liu, Shengying Liang, Fen Yang, Yuliang Sun, Lidan Niu, Yakun Ren, Hongmei Wang, Yanan He, Jiang Du, Jun Yang, Juntang Lin

**Affiliations:** 1 Stem Cell and Biotherapy Technology Research Center, College of Life Science and Technology, Xinxiang Medical University, Xinxiang, 453003 China; 2 Henan Key Laboratory of Medical Tissue Regeneration, NO 601, East of JinSui Road, Xinxiang City, 453003 Henan Province China; 3 College of Biomedical Engineering, Xinxiang Medical University, Xinxiang, 453003 China; 4 The First Affiliated Hospital of Xinxiang Medical University, NO 88, JianKang Road, Weihui, Xinxiang City, 453100 Henan Province China

**Keywords:** Endometriosis, Endometrial mesenchymal stem cells, Endometriotic mesenchymal stem cells, Angiogenesis, Immunomodulation

## Abstract

**Background:**

Research into the pathogenesis of endometriosis (EMs) would substantially promote its effective treatment and early diagnosis. However, the aetiology of EMs is poorly understood and controversial despite the progress in EMs research in the last several decades. Currently, accumulating evidence has shed light on the importance of endometrial stem cells (EnSCs) residing in the basal layer of endometrium in the establishment and progression of endometriotic lesions. Therefore, we aimed to identify the differences between EnSCs isolated from the ectopic lesions of EMs patients (EnSC-EM-EC) and EnSCs isolated from eutopic endometrium of control group (EnSC-Control). We further performed preliminary exploration of the potential signalling pathways involved in the above abnormalities.

**Methods:**

EnSC-EM-EC (*n* = 12) and EnSC-Control (*n* = 13) were successfully isolated. Then, the proliferative capacity, migratory capacity and angiogenic potential of EnSCs were evaluated by conventional MTT assay, flow cytometry, wound healing assay, transwell assay, tube formation assay and chick embryo chorioallantoic membrane assay respectively. The expression of 11 angiogenesis-associated biological factors and 11 cytokines secreted by EnSCs and 17 adhesion molecules expressed on EnSCs were determined by protein array assays respectively. Differentially expressed genes (DEGs) between EnSC-EM-EC and EnSC-Control were analysed by RNA-sequence.

**Results:**

EnSC-EM-EC exhibited unique biological characteristics, including prolonged mitosis, enhanced migratory capacity and enhanced angiogenic potential. Greater amounts of angiogenic factors (especially VEGF and PDGF) were secreted by EnSC-EM-EC than by EnSC-Control; however, the distinct profiles of cytokines secreted by EnSC-EM-EC and adhesion molecules expressed by EnSC-EM-EC require further investigation. A total of 523 DEGs between EnSC-EM-EC and EnSC-Control were identified and analysed using the KEGG and Gene Ontology databases.

**Conclusions:**

Our results not only improve the understanding of EMs but also contribute to the development of EnSC-EM-EC as a tool for EMs drug discovery. These cells could be of great help in exploiting promising therapeutic targets and new biomarkers for EMs treatment and prognosis.

## Background

Endometriosis (EMs) is an oestrogen-dependent, progesterone resistant, inflammatory, benign gynaecological disorder that is commonly observed in women of reproductive age (5–15% of reproductive-age women and 20–50% of women with chronic pelvic pain and/or infertility) [1–3]. EMs is histologically characterized by the presence of endometrial-like tissues (composed of both glandular and stromal tissue) outside the uterus, and this tissue undergoes cyclic regeneration and shedding processes similar to those of the normal endometrium. Consequently, these ectopic endometrial-like tissues cause not only chronic, intolerable pain (dysmenorrhea, dyspareunia and pelvic pain) but also subfertility or infertility, and these two main clinical symptoms of EMs profoundly affect patient quality of life [4, 5]. However, current treatment strategies, including surgical procedures, medical therapy or a combination of both, fail to prevent high recurrence of EMs and produce serious side effects that negatively affect the fertility and reproductive health of women [4, 6]. Therefore, the importance and urgency of understanding EMs pathogenesis is underscored by health concerns everywhere, in order to explore more definitive therapies.

To date, the aetiology of EMs is still poorly understood and sometimes controversial despite the progress in EMs research in the last several decades [4, 7–10]. In addition to the theories of coelomic metaplasia, embryonic cell rest, induction and lymphatic and vascular dissemination, the implantation theory, which is based on retrograde menstruation, is the most widely accepted aetiological explanation of EMs; it focuses on the pathogenesis of peritoneal and ovarian EMs, postulating that retrograde menstruation delivers sloughed endometrial fragments to the ectopic locations where they implant and grow [11, 12]. However, most women experience retrograde menstruation, but only appropriately 10% of reproductive-aged women develop EMs, suggesting that additional mechanisms are involved. Thus far, accumulating preclinical and clinical evidence indicates that the interactions among genetic, endocrine, immunological and environmental factors are likely to be involved in the enigmatic pathogenesis of EMs [1, 7, 13].

However, no matter which kind of theory was postulated to explain the EMs pathogenesis, the core aim is to clarify the location at which the ectopic endometrial-like tissues originate, and that is why the retrograde menstruation-derived implantation theory is widely accepted. First, EMs is rare in non-menstruating species; second, ectopic lesions can be successfully formed in animal models after implantation of unfractionated xenogeneic human endometrium; and third, endometrial mesenchymal stem cells (EnSCs) exist in the basal layer of the endometrium and in menstrual debris [8, 14]. In particular, the confirmation that EnSCs reside in ectopic lesions of EMs patients not only improved and promoted the implantation theory but also strongly suggested a prominent role for EnSCs in contributing to the establishment of ectopic lesions and also the progression of the EMs [15]. Currently, several excellent reviews detail the biological characteristics of EnSCs and their potential role in EMs pathogenesis [8, 16–18]. Therefore, the present study aimed to determine the biological characteristics, especially the angiogenic potential, of EnSCs isolated from ectopic lesions of EMs patients (EnSC-EM-EC) compared with those of EnSCs isolated from eutopic endometrium of control individuals (EnSC-Control). Furthermore, protein array assays and RNA sequencing (RNA-seq) were performed to identify the potential candidate genes or proteins that trigger the abnormal activity of EnSC-EM-EC, as these genes could greatly assist the identification of promising therapeutic targets and new therapeutic and prognostic biomarkers in EMs.

## Materials and methods

### Human specimens

This study was approved by the Ethics Committee of the Xinxiang Medical University. All human-derived specimens were collected under sterile conditions from the First Affiliated Hospital of Xinxiang Medical University and immediately transported to the laboratory for further processing within 8 h of detachment. All experimental procedures for human-derived specimens, EnSCs and human umbilical cord venous endothelial cells (HUVECs) were carried out in accordance with the approved guidelines. Written informed consent was obtained from all patients and donors prior to tissue collection, and all patients and donors provided consent for the use of their tissue samples for scientific research. Umbilical cord tissues were collected from healthy mothers after full-term delivery. Ectopic lesions from EMs patients (experimental group, *n* = 12, age range 25–47 years) and eutopic endometrium from uterine fibroid patients without EMs (control group, *n* = 13, age range 36–53 years) were obtained from patients who underwent standard gynaecological surgery and had pathologically confirmed results. To decrease the likelihood of using incorrectly identified tissue, only ultrasound-confirmed early proliferative (1–3 days after menstruation) endometrial fragments were selected for EnSC isolation, and no participating patient received hormone therapy for at least 3 months before surgery and had any other pelvic pathology.

### Fertilized eggs, cells and antibodies

Fresh fertilized eggs were purchased from a local farm (Xinxiang, China). All experimental protocols were approved by the Animal Research Committee of Xinxiang Medical University according to the Chinese Council on Animal Care guidelines. HUVECs, EnSC-Control (EnSCs isolated from eutopic endometrium of uterine fibroid patients) and EnSC-EM-EC (EnSCs isolated from ectopic endometrium of EMs patients) isolation are described in detail below. Details of all antibodies used in this study are provided in the Supplementary Information (Table S1).

### Isolation and culture of EnSCs

EnSC isolation was performed as described previously [19]. In brief, the fresh endometrial tissue samples were rinsed with PBS and minced into small pieces (1 mm^3^) with sterile scissors. The tissue fragments were digested with type I collagenase [1 mg/mL (Sigma, USA), diluted in DMEM/F12 medium (HyClone, USA)] for 60 min at 37 °C with shaking every 10 min, and the tissue digests were successively filtered through 100 μm and 40 μm cell strainers (Corning, New York, USA). Then, the cell suspension was washed twice with PBS and gently resuspended in growth medium [DMEM/F12 supplemented with 10% FBS (Gibco, USA) and 1% penicillin/streptomycin (P/S, Life Technologies, USA)], seeded into 25 cm^2^ cell culture flasks and incubated at 37 °C in a humidified atmosphere containing 5% CO_2_. After 2 days of incubation, the non-adherent cells were washed away leaving behind the adherent cells that were growing as fibroblastic cells in clusters. The medium was replaced every 3 days. At 80–90% confluence (P0), the cells were detached by treatment with 0.25% trypsin/1 mM EDTA and subcultured into new flasks at a ratio of 1:2. Finally, all EnSC samples were cryopreserved using standard methods in complete growth medium containing 10% DMSO for the subsequent experiments.

### Immunofluorescence analysis

Cells were fixed with 4% PFA for 20 min and permeabilized with 0.05% Triton X-100 for 10 min. Nonspecific binding was blocked with 5% goat serum (Beyotime, China) for 30 min. Primary antibodies were added, and the cells were incubated at 4 °C overnight. Alexa Fluor 488-conjugated goat anti-mouse or anti-rabbit secondary antibodies were incubated with the cells at 37 °C for 1 h. Cell nuclei were stained with DAPI. Finally, the cells were observed and imaged under an inverted fluorescence microscope (Leica, Germany). The fluorescence intensity was quantified with ImageJ software based on 10 randomly collected images, and the mean fluorescence intensity, integrated fluorescence density and percentage of the area exhibiting fluorescence were determined.

### Immunophenotyping analysis

P3 EnSC-Control and EnSC-EM-EC were used for immunophenotyping analysis. The following mouse anti-human monoclonal antibodies were used: FITC-conjugated CD29, CD73, CD90, HLA-ABC, HLA-DR, CD34, and CD45; and PE-conjugated CD105. As negative controls, isotype PE- and FITC-conjugated IgG were used. The cell suspension (1 × 10^6^ cells) was washed twice with PBS and incubated with the monoclonal antibodies at 4 °C in the dark for 30 min. The samples were washed with PBS and analysed in a Cytomics FC 500 MPL cytometer (Beckman Coulter, USA).

### In vitro multilineage differentiation assays

Adipogenic and osteogenic differentiation were performed and analysed as reported [20]. In brief, for differentiation assays, P3 EnSCs were suspended in growth medium, seeded at a density of 2 × 10^4^ cells/well in a 6-well plate and grown to confluence. Subsequently, the growth medium was replaced with adipogenic differentiation medium (growth medium + 1 μmol/L dexamethasone + 10 μg/ml recombinant human insulin + 200 μmol/L indomethacin + 0.5 mmol/L IBMX, for 14 days) or osteogenic differentiation medium (growth medium + 0.1 μmol/L dexamethasone + 0.05 mmol/L ascorbic acid + 10 mmol/L β-glycerophosphate, for 21 days), and the induction medium was replaced every 3 days. Control cells were cultured in growth medium. At the end of the induction period, the cells were washed and fixed. Adipogenic differentiation was confirmed by Oil red O staining; osteogenic differentiation was confirmed by Alizarin red staining.

### MTT assay

To assess the metabolic activity of EnSC-Control and EnSC-EM-EC, P3 cells were suspended in growth medium and seeded at a concentration of 2.5 × 10^4^ cells/ml in 96-well plates. After incubation at 37 °C in a humidified atmosphere containing 5% CO_2_ for 1, 3, 5 and 7 days, metabolic activity was evaluated with conventional MTT assay. Absorbance values were measured at 490 nm using an ELISA reader SpectraMax® i3 (Molecular Devices, USA).

### Cell cycle analysis

To assess the cell cycle status, P3 EnSC-Control and EnSC-EM-EC (1 × 10^6^ cells) were detached, pelleted and fixed with ice-cold 70% ethanol at 4 °C overnight in accordance with the instructions of the cell cycle analysis kit (Beyotime Biotechnology, China). After two washes with PBS, the cells were incubated with 50 μl of diluted RNase solution at room temperature for 15 min. Next, 500 μl of diluted propidium iodide solution was added for staining, and the cells were incubated in the dark for 30 min at 37 °C. Finally, the cell cycle phase distribution (G0/G1, S and G2/M phase) was analysed in a Cytomics FC 500 MPL cytometer. Data are shown as the mean ± SD of three independent biological samples per group.

### Wound healing assay

For the wound healing assay, P3 EnSCs in complete growth medium were seeded in six-well plates. When the cells were 100% confluent, a straight liner scratch was made in the monolayer using a sterile 200 μl pipette tip. Then, the cells were washed with PBS to remove detached cells and incubated with serum-free DMEM/F12 medium for 72 h. Images were acquired at different time points under an inverted microscope. The cells that migrated into the wounded area were quantified with ImageJ analysis software, and data are shown as the mean ± SD of three independent biological samples per group.

### Transwell assay

For the transwell migration assay, P3 EnSCs (25 × 10^3^ cells) suspended in serum-free DMEM/F12 were seeded in the upper chamber of the insert (8 μm pore diameter, Corning) in 24-well plates and were allowed to migrate towards growth medium in the lower chamber (DMEM/F12 supplemented with 10% FBS) at 37 °C with 5% CO_2_. After 24 h, the non-migrated cells in the upper chamber of the inserts were carefully scraped off with a wet cotton swab, and the inserts were subsequently washed with PBS. Then, the cells that migrated to the lower surface of the insert were fixed with 4% paraformaldehyde for 30 min and stained with crystal violet. The stained cells were quantified with ImageJ analysis software, and the data are shown as the mean ± SD of three independent biological samples per group.

### Preparation of conditioned medium from EnSCs

Two million P3 EnSCs were seeded into 75 cm^2^ plastic cell culture flasks and cultured for 12 h. Then, the growth medium was replaced with fresh medium (DMEM/F12 + 2% FBS + 1% P/S). Cells were cultured for another 48 h, and the conditioned medium (CM) was collected, filtered through a 0.22-μm filter and stored at − 80 °C.

### Tube formation assay

The tube formation assay was carried out as previously described [21]. HUVECs were isolated, identified and cryopreserved by our research group as described in the supplementary methods section and Figure S6. In brief, for the tube formation assay, P3 HUVECs were trypsinized and resuspended in CM from EnSCs prepared as described above and were then gently seeded at a density of 6 × 10^4^ cells/well in triplicate in 96-well plates pre-coated with growth factor reduced Matrigel (BD Biosciences, USA) according to the manufacturer’s instructions. The cells were incubated at 37 °C with 5% CO_2_ for 6 h, and images of tube-like structures in each well were captured using an inverted microscope (Leica, German). Furthermore, the total length, branch length, node number, branch number and junction number in the scanned images were observed and quantitated with ImageJ software. Data are shown as the mean ± SD of three independent biological samples per group.

### Chick embryo chorioallantoic membrane (CAM) assay

A CAM assay was used to evaluate the in vivo angiogenic potential of EnSCs as previously described with a minor improvement, as shown in Figure S7 [22]. Eggs were collected and were then randomly separated into three groups (*n* = 10): the control group (addition of 200 μl of PBS to the centre of the silicon ring), the EnSC-Control treatment group (addition of 2 × 10^6^ cells in 200 μl of PBS) and the EnSC-EM-EC treatment group (addition of 2 × 10^6^ cells in 200 μl of PBS). Furthermore, the opening window on the shell was sealed with tape to prevent dehydration and infection. All procedures were performed under sterile conditions. After 3 days of incubation (day 10), the tape covering the opening was removed and the CAMs were imaged in ovo under a stereomicroscope. Then, the vascular area and branch number in the images were observed and quantitated with ImageJ software. Data are shown as the mean ± SD of five independent biological samples per group.

### Protein array assays

Angiogenesis and inflammation arrays (AAH-CUST-G1, RayBiotech, Norcross, GA, USA) were used according to the manufacturer’s instructions to measure the expression levels of 11 angiogenesis-associated biological factors and 11 cytokines in EnSC-derived CM that had been concentrated tenfold by ultrafiltration (*n* ≥ 5). Adhesion molecule arrays (GSH-CAM-1, RayBiotech, Norcross, GA, USA) were used to measure the expression levels of 17 adhesion molecules on P3 EnSCs (*n* ≥ 6). Positive signals were captured on glass chips using a laser scanner (InnoScan 300 Microarray Scanner; Innopsys, Carbonne, France), and the observed fluorescence intensities were normalized to those of the internal positive controls.

### RNA-seq

P3 EnSCs (2 × 10^6^ cells) were collected and delivered to the Beijing Genomics Institute (BGI, Wuhan, China) on dry ice. The following procedures, including total RNA extraction, oligo (dT) magnetic bead enrichment and reverse transcription with N6 random primers, were performed by BGI to ensure the high quality of the samples (RIN range 9.6–10, average 9.72 ± 0.14). Then, sequencing was performed by BGI on the BGISEQ-500 platform, generating an average of 23,754,820 raw sequencing reads and 23,694,414 clean reads after filtering low-quality reads. The high-quality reads were aligned to the human reference genome using the HISAT/Bowtie2 tool. The expression level of each of the gene was normalized to fragments per kilobase of exon model per million mapped reads (FPKM) method using RNA-seq by Expectation Maximization (RSEM). Then, differentially expressed genes (DEGs) between EnSC-Control (*n* = 5) and EnSC-EM-EC (*n* = 6) were analysed by the NOISeq method with default criteria (fold change ≥ 2 and diverge probability ≥ 0.8), and the screened DEGs were further subjected to Gene Ontology (GO) and pathway enrichment analysis based on the KEGG database.

### Western blot analysis

EnSCs (*n* = 3) were washed with cold PBS and resuspended in RIPA buffer (CST, Technology, #9806) supplemented with a protease inhibitor cocktail (CST, Technology, #5871). Cell lysates (20 μg) were electrophoresed through polyacrylamide gels. Proteins were transferred to PVDF membranes, which were blocked with 5% non-fat milk in PBS for 1 h and then incubated separately with primary antibodies overnight at 4 °C. Membranes were then incubated with HRP-conjugated secondary antibodies. GAPDH was used as the internal control. Immunoreactions were detected using enhanced chemiluminescence reagent in an Amersham Imager 600 (GE Healthcare Life Sciences).

### Statistical analysis

The results are representative of at least three independent experiments and presented as the mean ± SD. To determine statistical significance, Student’s *t* test was used for comparisons between two groups; one-way ANOVA followed by Dunnett’s test was used for comparisons among ≥ 3 groups. *P* < 0.05 was considered to be statistically significant.

## Results

### Identification of EnSCs

**Fig. 1:**
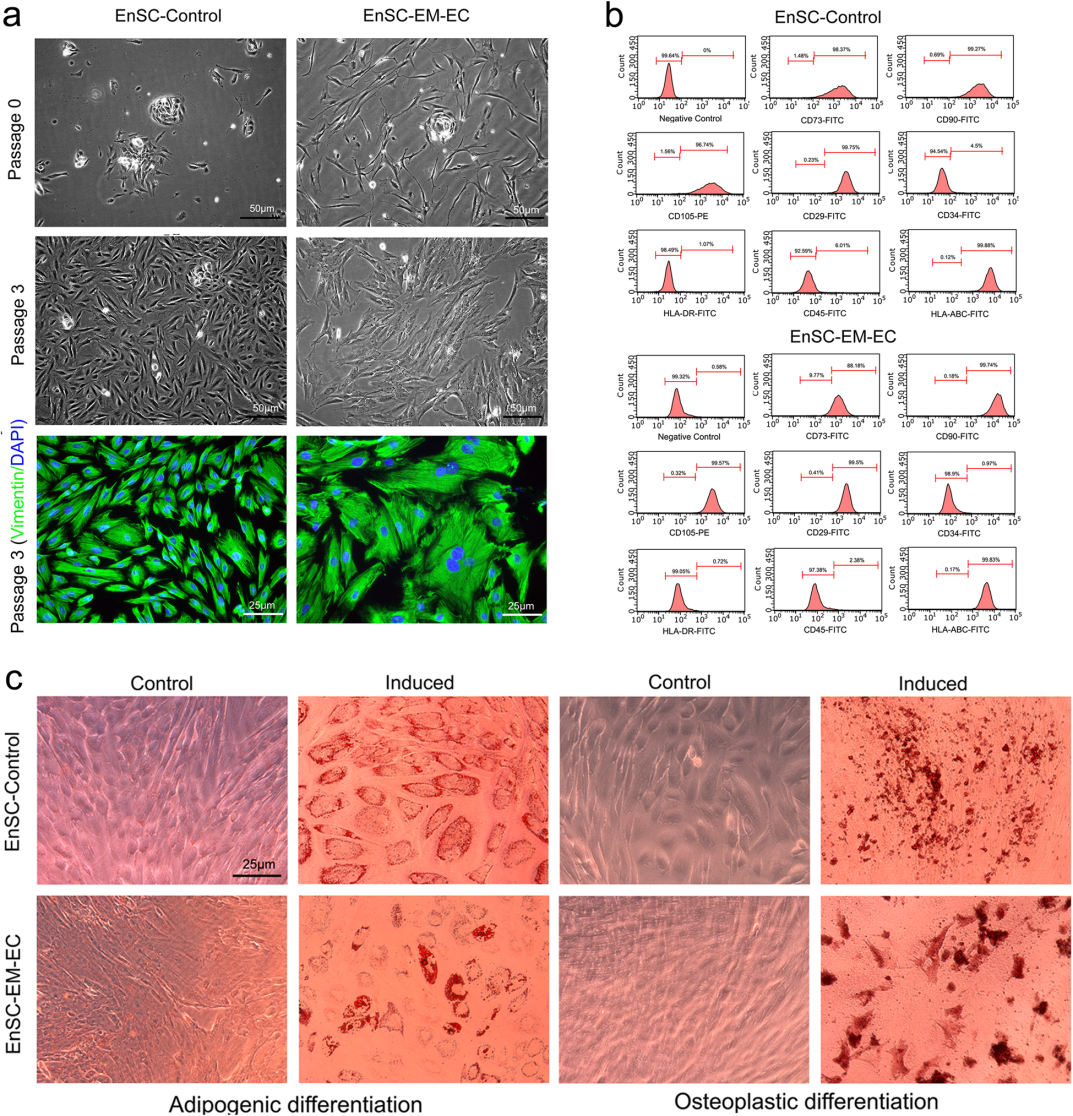
Isolation and identification of EnSCs. **a** Morphology of EnSCs. P0 and P3 EnSC-Control showed a typical spindle-shaped, fibroblast-like morphology with a radial or helical growth pattern, but EnSC-EM-EC showed an irregular morphology instead of a spindle-shaped, fibroblast-like morphology. Furthermore, the expression of vimentin in EnSC-Control and EnSC-EM-EC cells are examined by conventional immunofluorescence. Representative images are shown. **b** Phenotype of EnSCs. Both EnSC-Control and EnSC-EM-EC were positive for the expression of typical ASC markers (CD29, CD73, CD90 and CD105) and HLA-ABC and negative for the expression of haematopoietic stem cell markers (CD34 and CD45) and HLA-DR. **c** Conventional adipogenic and osteogenic differentiation were induced, and differentiation was visualized as positive Oil red O and Alizarin red staining. Scale bar, 25 μm

During primary culture, EnSCs exhibited a colony-like morphology, which was clearly observed in both EnSC-Control and EnSC-EM-EC (Fig. 1a). As previously reported, subcultured EnSC-Control demonstrated growth characteristics typical of ASCs (a spindle-shaped, fibroblast-like morphology with a radial or helical growth pattern); however, subcultured EnSC-EM-EC exhibited an irregular morphology instead of a typical spindle-shaped, fibroblast-like morphology. Additionally, EnSC-EM-EC isolated from individual patients were consistent and showed similar morphological characteristics (Figure S1). Subsequently, both EnSC-Control and EnSC-EM-EC were found to express vimentin, a classical mesodermal skeleton protein, suggesting that EnSCs were derived from mesoderm. Third, flow cytometric analysis of P3 EnSCs demonstrated that both EnSC-Control and EnSC-EM-EC were positive for CD29, CD73, CD90, CD105 and HLA-ABC expression but negative for CD34, CD45 and HLA-DR expression (Fig. 1b). Finally, as shown in Fig. 1c, the results of multilineage differentiation assays confirmed that the EnSCs can undergo adipogenic and osteogenic differentiation after treatment with induction medium.

### EnSC-EM-EC exhibited inferior metabolic activity and a prolonged mitotic phase

**Fig. 2:**
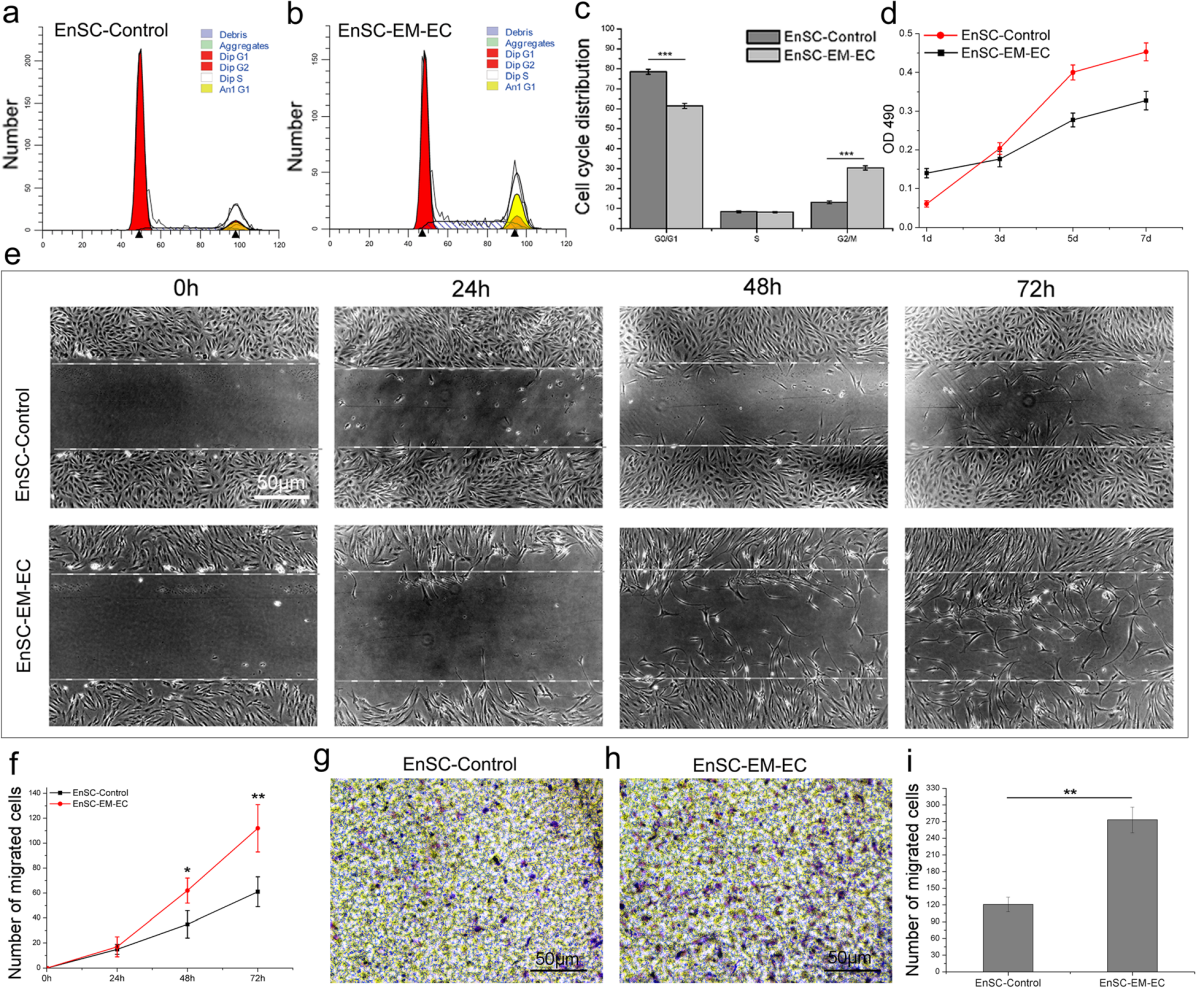
Proliferative and migratory capacities of EnSCs. **a**, **b** P3 EnSC-Control and EnSC-EM-EC were used to assess the cell cycle status as directed by the kit instruction. Representative flow cytometric results are shown. **c** Quantification of flow cytometric cell cycle assay results. **d** P3 EnSC-Control and EnSC-EM-EC were seeded into 96-well plates, and the proliferative capacity was determined by a conventional MTT assay at the indicated time points. **e** P3 EnSC-Control and EnSC-EM-EC cells were seeded into 6-well plates and cultured to 100% confluence. Then, wound healing assay was performed to examine the number of cells migrating into the wounded area at the indicated time points. Representative images are shown. Scale bar, 50 μm. **f** Quantification of the wound healing assay results. **g**, **h** P3 EnSC-Control and EnSC-EM-EC in serum-free culture medium were seeded in the upper chamber of inserts in 24-well plates and were allowed to migrate towards growth medium supplemented with 10% FBS for 24 h. Then, the cells that migrated to the lower surface of the insert were fixed and stained with crystal violet. Representative images are shown. **i** Quantification of the transwell assay results

Cell cycle analysis (Fig. 2a–c) demonstrated both a significant increase in mitotic cells (*P* < 0.01) and a decrease in cells in the resting phase (*P* < 0.05) in EnSC-EM-EC, suggesting the superior proliferative capacity of EnSC-EM-EC compared with EnSC-Control. However, based on our experience and further MTT results (Fig. 2d), the proliferation rate and metabolic activity of EnSC-EM-EC were actually lower than those of EnSC-Control seeded at the same density; moreover, the metabolic activity of EnSC-EM-EC was significantly decreased at days 5 and 7 (*P* < 0.05) under standard culture conditions (37 °C, 5% CO_2_).

### EnSC-EM-EC exhibit superior migration capacity in vitro

To examine the difference in the migratory capacity between EnSC-EM-EC and EnSC-Control, wound healing and transwell assays were performed. Our results confirmed the previous findings, demonstrating the superior migratory capacity of EnSC-EM-EC. A significantly higher number (Fig. 2e, f) of EnSC-EM-EC than EnSC-Control had migrated into the wounded area after 48 h (*P* < 0.05) and 72 h (*P* < 0.01). Furthermore, EnSC-EM-EC exhibited an enhanced migratory capacity in the transwell assay, as evidenced by the higher number of cells than migrated through the insert to the lower surface (*P* < 0.01).

### EnSC-EM-EC exhibited enhanced angiogenic potential

**Fig. 3:**
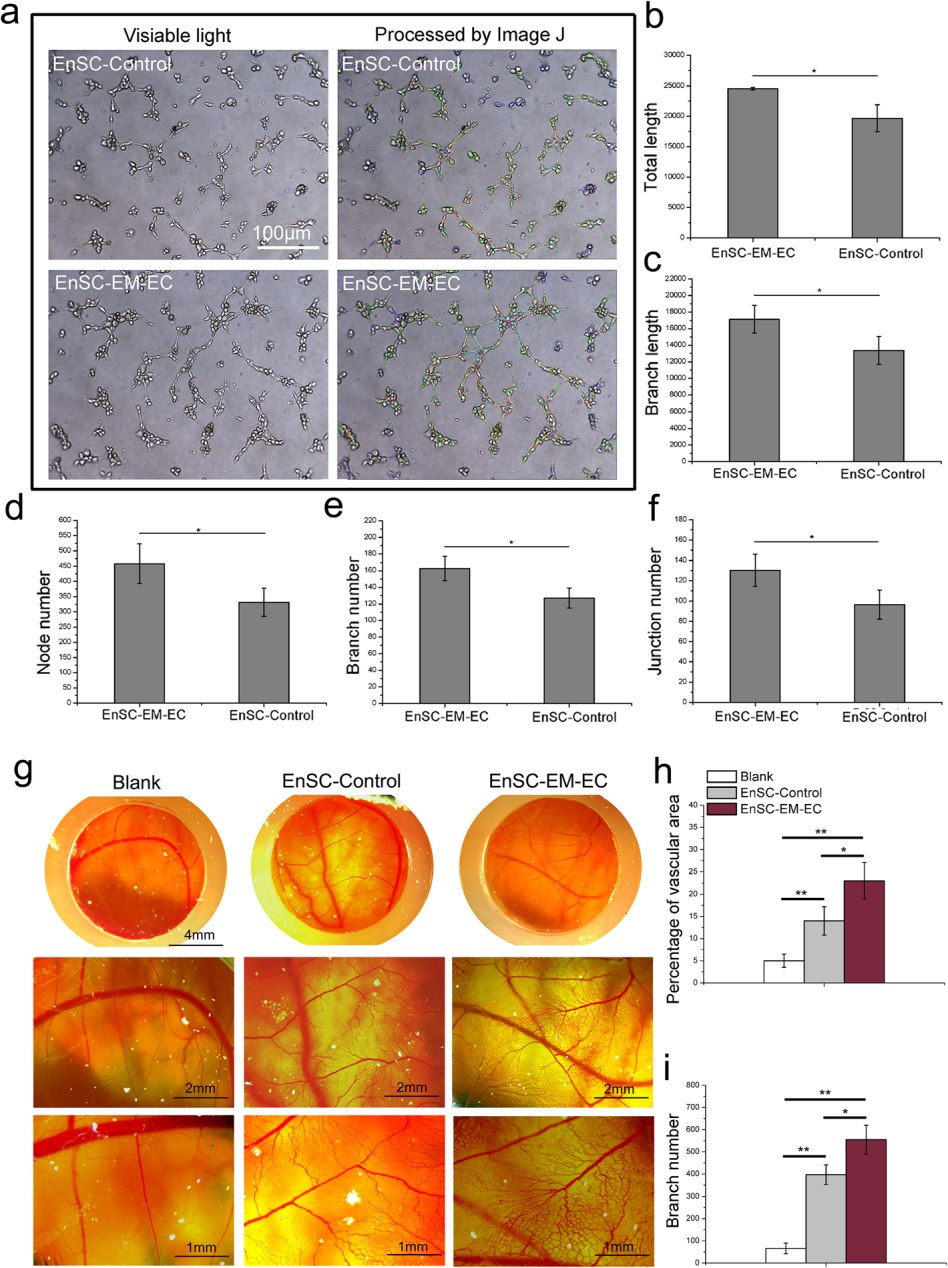
Angiogenic potential of EnSCs. **a** P3 HUVECs were suspended in CM from EnSC-Control or EnSC-EM-EC prepared as described in the “Materials and methods” section and were seeded into 96-well plates pre-coated with growth factor reduced Matrigel for 6 h. The tube-like structures were then imaged under an inverted microscope and analysed with ImageJ. **b**–**f** The total length, branch length, node number, branch number and junction number in the images of tube-like structures were quantitated with ImageJ. **g** P3 EnSC-Control or EnSC-EM-EC suspended in 200 μl of PBS were seeded into a silicone loop placed on top of the growing CAM between mature blood vessels and incubated for 3 days. Images were acquired in ovo under a stereomicroscope. **h**, **i** The vascular area and branch number in the images were observed and quantitated with ImageJ

The results of the successful tube formation assay (Fig. 3a–f) demonstrated that compared with CM from EnSC-Control, CM from EnSC-EM-EC significantly promoted the formation of tube-like structures, as evidenced by the greater total length, branch length, node number, branch number and junction number (*P* < 0.05). Moreover, as shown in Fig. 3g–i, the results of the chick embryo CAM assay confirmed the enhanced pro-angiogenic potential of EnSC-EM-EC, as evidenced by the significantly greater vascular area and branch number in EnSC-EM-EC-treated CAMs (*P* < 0.05).

### EnSCs-EM-EC released greater amount of angiogenic factors than control cells

**Fig. 4:**
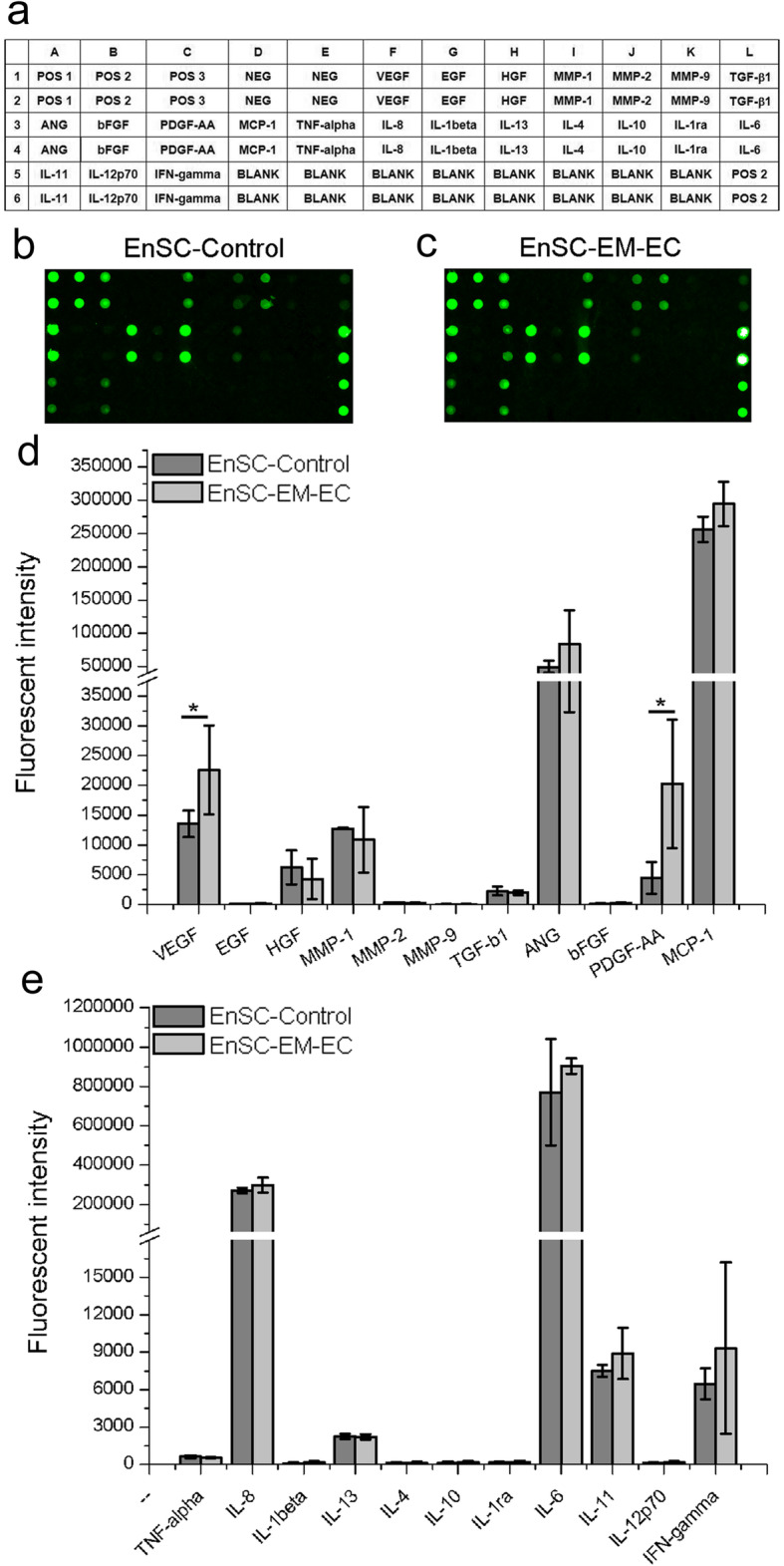
Paracrine secretion of biological factors into CM from EnSCs. **a** Set of biological factors examined. **b**, **c** Representative protein array images are shown. **d**, **e** Fluorescence intensity of the indicated biological factors

The paracrine effect plays a critical role in the behaviour of mesenchymal stem cells (MSCs). Thus, a protein array assay was performed to quantify the associated angiogenic and immunomodulatory factors secreted into the CM by EnSCs (Fig. 4 and Figure S2). As shown in Fig. 4d, e, both EnSC-Control and EnSC-EM-EC exhibited a similar paracrine profile for the examined biological factors. In addition, classical pro-angiogenic factors (VEGF, HGF, ANG, PDGF and MMP-1) were expressed at high or moderate levels in CM from EnSCs, as were several inflammatory cytokines (MCP-1, IL-6, IL-8, IL-11 and IFN-γ). Furthermore, compared with EnSC-Control, EnSC-EM-EC secreted greater amounts of VEGF and PDGF (*P* < 0.05) into the CM, but inflammatory cytokine secretion did not differ between the cells.

### Expression of adhesion molecules on EnSCs

**Fig. 5:**
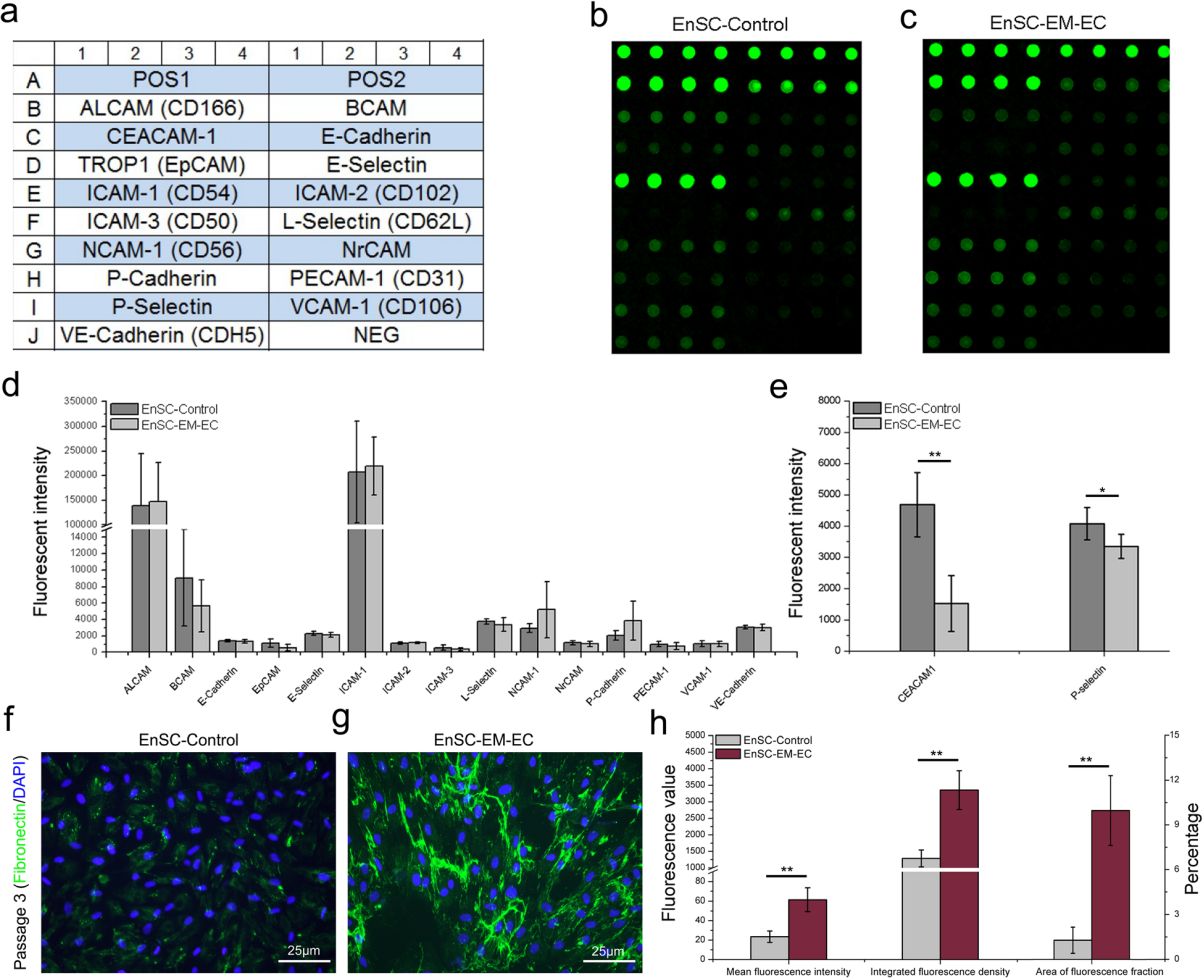
Expression of adhesion molecules on EnSCs. **a** Set of adhesion molecules examined. **b**, **c** Representative protein array images are shown. **d**, **e** Fluorescence intensity levels of the indicated adhesion molecules. **f**, **g** Expression of fibronectin in P3 EnSC-Control and EnSC-EM-EC cells was examined by conventional immunofluorescence. Representative images are shown. **h** The mean fluorescence intensity, integrated fluorescence density and percentage area of fluorescence were quantitated in the images with ImageJ

The adhesion molecule expression profile on EnSCs was also determined via a protein array assay (Figure S3). As shown in Fig. 5a–e, the 17 adhesion molecules exhibited a similar expression profile in both cell types. In addition to expressing extremely high levels of ALCAM and ICAM-1, EnSCs expressed moderate levels of BCAM, CEACAM-1, L-selectin, NCAM, P-selectin and VE-cadherin. Moreover, the expression levels of CEACAM-1 and P-selectin on EnSC-EM-EC were significantly decreased compared with those on EnSC-Control (*P* < 0.05). Simultaneously, the expression of fibronectin in EnSCs was examined by conventional IF. Unlike EnSC-Control, EnSC-EM-EC exhibited a significant increase in fibronectin expression (Fig. 5f–h, *P* < 0.01), suggesting the enhanced adhesion capacity of EnSC-EM-EC.

### Five hundred twenty-three DEGs between EnSC-Control and EnSC-EM-EC and the pathways enriched with these genes were identified and partially confirmed

**Fig. 6:**
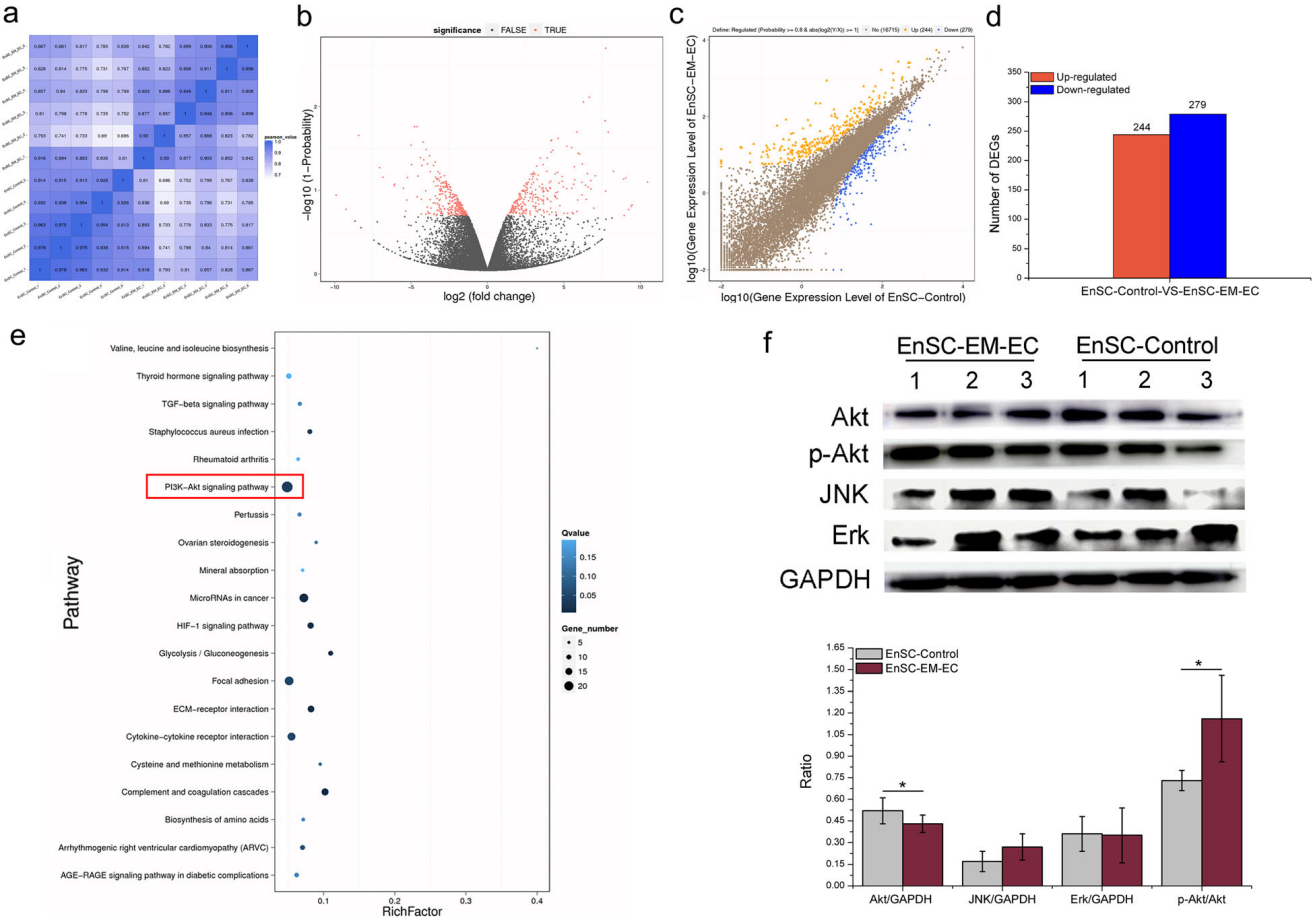
The DEGs between EnSC-Control and EnSC-EM-EC and the pathways enriched with these DEGs. **a** Heat map of correlation coefficients for the analysis of EnSC-Control and EnSC-EM-EC. The gradient colour barcode at the top right indicates the minimum value in white and the maximum in blue. If one sample is highly similar to another sample, the value of the correlation coefficients is very close to 1. **b** Volcano plot of all genes expressed between EnSC-Control and EnSC-EM-EC. The red dots indicate significant DEGs that passed the screening threshold, and the black dots indicate nonsignificant DEGs. **c** Scatter plots of all genes expressed in EnSC-Control and EnSC-EM-EC. The blue rhombi indicate downregulated genes, the orange triangles indicate upregulated genes and the brown dots indicate genes with unaltered expression. The screening threshold is shown above the legend. **d** Statistics of DEGs between EnSC-Control and EnSC-EM-EC. The blue bars denote downregulated genes, and the orange bars denote the upregulated genes. **e** Pathway enrichment statistics for DEGs between EnSC-Control and EnSC-EM-EC. The rich factor indicates the ratio of the number of differentially expressed gene annotated in a pathway term to the number of all genes annotated in that pathway term. A higher rich factor indicates higher enrichment. The *Q* value is the corrected *p* value (range 0–1) and a lower *Q* value indicates higher enrichment. Only the top 20 enriched pathway terms are shown. **f** Conventional WB was used to identify the key roles of PI3K/Akt signalling pathways. The grayscale value of the band representing each targeted protein was quantitated with ImageJ

Furthermore, unbiased transcriptome profiling was performed via RNA-seq to reveal the potential genes and signalling pathways responsible for the significant difference in the biological characteristics of EnSC-Control and EnSC-EM-EC, as mentioned above. As shown in Fig. 6a–d, of the 523 screened DEGs (Table S2), 244 were upregulated, and the other 279 were downregulated in EnSC-EM-EC. Subsequently, GO annotation analysis was performed on the identified DEGs, and Web Gene Ontology Annotation Plot software was used to perform GO functional classification. The distribution of the DEG functions at the macro level is shown in Figures S4 and S5. Next, we subjected the identified DEGs to specific pathway enrichment analysis based on the KEGG database. The top 20 KEGG pathways enriched with the DEGs are shown in Fig. 6e. Additionally, to gain an in-depth understanding of Fig. 6e, 8 of the most relevant pathways were selected based on general knowledge about EMs. The DEGs referenced in these pathways are presented in Table S3. In addition, based on the pathways enriched with the DEGs and published reports, we further examined the core component of the PI3K-Akt signalling pathway at the protein level. As shown in Fig. 6f, consistent with the results at the gene level, the Akt expression was significantly downregulated, and Akt phosphorylation was significantly upregulated in EnSC-EM-EC, but the expression of Erk and JNK did not differ markedly between EnSC-Control and EnSC-EM-EC.

## Discussion

EMs is defined as a benign disease that is unlikely to endanger the life of patients. However, both the clinical symptoms triggered by EMs, including dysmenorrhea, pelvic pain, dyspareunia and infertility, and the effects resulting from the high rate of recurrence after surgical and/or medical treatment not only severely affect the physical and mental health of patients, but also result in heavy social and economic burdens [23–25]. To date, although various theories have been proposed to explain the pathogenesis of EMs, the aetiology of the disease remains elusive and somewhat controversial despite decades of clinical experience and research [4, 7–10]. All theories (the coelomic metaplasia, embryonic cell rest, induction and lymphatic and vascular dissemination and implantation theories) aim primarily to identify the seeding cells that form the final ectopic lesions. Therefore, since the first demonstration of the existence of EnSCs (endometrial epithelial and stromal cells) in the endometrium in 2004, the theory of EnSCs has provided a new perspective to the pathogenesis of EMs [16–18, 26].

### Existence of EnSCs in endometriotic lesions

In the past decade, the existence of EnSCs has been extensively confirmed and broadly accepted. A full review of EnSCs is beyond the scope of this study, and the reader cab refers to the publication by Gargett et al. for a comprehensive overview of their biological characteristics, therapeutic application and potential pathogenic role in gynaecological disease [14]. Similarly, high telomerase activity in human endometriotic lesions was first reported in 2007, and a subsequent study demonstrated significant increases in the mRNA and protein levels of stemness-related markers, including *UTF1*, *TCL1*, *ZFP42*, SALL4 and Musashi-1, in human endometriotic lesions compared to control endometrium [27–29]. Furthermore, in a rat model of EMs, ectopic endometrium exhibited significantly higher gene expression of *Oct4* and *SSEA1* than those in control endometrium [30]. These findings strongly suggest that EnSCs are present in ectopic lesions. In 2011, Chan et al. demonstrated that, as expected, ovarian endometriotic cysts contain a subset of epithelial and stromal progenitor cells displaying somatic stem cell properties (colony-forming activity, self-renewal capacity and multipotency), although the colony-forming activity of these progenitor cells is lower than that observed in control women without EMs [31]. A recent study based on droplet digital PCR further indicated that the epithelial and stromal components involved in ectopic lesion formation in patients with EMs co-develop from independent progenitors rather than arise from a single or a few multipotent stem/progenitor cells [32].

### Enhanced migratory and angiogenic capacities of EnSC-EM-EC

Simultaneously accumulating evidence confirms that ectopic EnSCs are genetically and phenotypically different from their physiological analogues, and these cells display a more “aggressive” phenotype with higher proliferative, migratory, adhesive and angiogenic potentials and lower immunomodulatory capacity than eutopic EnSCs from women either with or without EMs [31, 33–35]. In accordance with the above findings, our results confirmed the enhanced migration of EnSC-EM-EC compared to that of EnSC-Control and demonstrated that both CM from EnSC-EM-EC or EnSC-EM-EC themselves significantly promoted angiogenesis. Furthermore, the results of protein array assays indicated that EnSCs cultured in vitro secreted greater amount of angiogenic factors (VEGF, HGF, ANG and PDGF-AA) than control cells. Moreover, the significant increase in the VEGF and PDGF-AA levels in CM from EnSC-EM-EC could partially explain the enhanced pro-angiogenic capacity of EnSC-EM-EC. Additionally, EnSC-EM-EC exhibited significantly upregulated expression of fibronectin, which is intimately involved in the development and remodelling of the vasculature [36]. In particular, fibronectin is often highly expressed around blood vessels during developmental and pathological angiogenesis [37], and circulating fibronectin has been shown to promote blood vessel formation and tumour growth through increased retention of VEGF in the tumour microenvironment [38].

However, the proliferation rate and metabolic activity of EnSC-EM-EC in this study were lower than those of EnSC-Control cultured under standard conditions (37 °C, 5% CO_2_), which is inconsistent with previous findings [34]. Theoretically, compared with the regenerative rate of normal endometrium (approximately 10 mm in each menstrual cycle), the growth rate of ectopic lesions in vivo is inhibited by the patients’ immune system and the limited supply of oxygen and nutrients. Therefore, we speculate that EnSC-EM-EC existed in ectopic lesions are accustomed to the harsh environment and that standard culture conditions (with a sufficient supply of oxygen and nutrients) are likely unfavourable for the growth of EnSC-EM-EC. This idea was partially supported by the abnormalities in the HIF signalling pathway confirmed by RNA-seq analysis of EnSC-EM-EC and EnSC-Control. Consequently, a reasonable postulation is that EnSC-EM-EC play a pivotal role in the persistence and recurrence of EMs.

### Immunomodulatory dysfunction of EnSC-EM-EC

In addition to angiogenesis, both inflammatory mediators (IL-1β, IL-6, IL-8, TNF-α and so on) and immune cells (neutrophils, macrophages, dendritic cells, NK cells, T cell subsets and so on) crucially contribute to the formation and progression of EMs, although whether inflammation is the cause or the result of EMs is uncertain [39]. Moreover, evidence indicates the association between inflammation and angiogenesis in endometriotic lesions, and inflammatory cells and endothelial cells can be activated by the same molecular events, further supporting this association [40, 41]. Interestingly, the immunomodulatory potential of menstrual blood-derived EnSCs has been verified; menstrual blood-derived EnSCs not only suppressed the lymphocyte proliferation and inflammatory cytokine secretion in an animal model of critical limb ischaemia but also modulated cytokine profiles and regulated immune cells to improve liver function and attenuate pathological changes in an animal model of acute liver injury [42–44] Therefore, to address whether the immunomodulatory potential of EnSC-EM-EC differs from EnSC-Control, Nair et al. reported in 2014 that EnSC-EM-EC exhibited an immune phenotype distinct from that of EnSC-Control; this phenotype included increased levels of TLRs, collectins and pro-inflammatory cytokines, which may be responsible for the reduced immunosuppressive activity of ectopic MSCs [35]. In contrast, in 2017, Abomaray et al. demonstrated that EnSC-EM-EC may be more immunosuppressive than EnSC-Control and may promote the activity of immunosuppressive M2 macrophages that support the growth of and reduce immunosurveillance in ectopic lesions [45]. Furthermore, in contrast to the findings of Nair et al., our in vitro results indicated that both types of EnSCs produced large amounts of pro-inflammatory cytokines (L-6, IL-8 and IFN-γ) and MCP-1 and that EnSC-EM-EC produced slightly but nonsignificantly greater amount of pro-inflammatory cytokines than EnSC-Control. Additionally, the low level of anti-inflammatory cytokine (IL-4, IL-10 and IL-13) secretion suggested the poor immunosuppressive capacity of EnSCs; this difference may be caused by the method of CM preparation and the different passages of cells used, which were not clearly described in previous studies. However, these in vitro results require further evaluation and verification because of the complex microenvironment in vivo.

Additionally, analysis of adhesion molecule expression demonstrated the extremely high expression of ALCAM and ICAM-1 on both types of EnSCs. ALCAM plays a role in the binding of T- and B cells to activated leukocytes, and ICAM-1 plays a role in the binding of leukocytes through binding to LFA-1(lymphocyte function-associated antigen 1). Subsequent studies in monocytes, endothelial cells and melanoma cells have also indicated the critical role of ALCAM in transendothelial monocyte migration, leukocyte adhesion and transmigration, and transition from local cell proliferation to tissue invasion [46]. In addition, knockdown of ALCAM in UM cells (MUM-2B) with high-ALCAM expression resulted in reduced motility and invasiveness [47]. Moreover, CD166^high^ UM cells (Mel 270) have a higher tumour transendothelial migration potential than CD166^low^ cells [48]. Furthermore, ICAM-1, a typical adhesion receptor, not only is most widely known for regulating leukocyte recruitment from circulation to inflammatory sites [49] but also is a master regulator of cellular responses during inflammation, injury resolution and tumorigenesis [50]. Further studies have also demonstrated that low expression levels of ICAM-1 expression critically impact adult stem cell-mediated immunosuppression [51, 52].

Interestingly, in 2016, Ahn et al. first reported that the mRNA expression level of carcinoembryonic antigen-related cell adhesion molecule 1 (CEACAM1) was significantly decreased in ectopic lesions of EMs patients compared to control endometrial tissue from patients without EMs [53]. Our results were consistent with these findings; the expression levels of CEACAM1 and P-selectin in EnSC-EM-EC were significantly reduced compared with those in EnSC-Control. CEACAM1 is an extensively studied cell surface molecule with established functions in angiogenesis and immunomodulation [54, 55]; further studies demonstrated that CEACAM1 expression is essential for HGF-induced migration of keratinocytes [56] and can either increase or decrease the migratory capacity of epithelial cells by interacting with distinct binding partners [57]. Moreover, P-selectin plays an essential role in the initial recruitment of leukocytes to the site of injury during inflammation [58]. Interactions between P-selectin and PSGL-1 play a role in platelet-mediated inflammation via the enhancement of neutrophil transmigration [59], and later studies indicated that P-selectin-mediated adhesion can activate chemokine- or platelet-activating factor-induced β2 integrins and further promote the release of chemokines from adherent leucocytes [60]. Consequently, the decreased expression of both CEACAM-1 and P-selectin in EnSC-EM-EC may explain the dysregulated inflammatory cytokine elevation and immune cell activation observed in EMs patients.

### Fibrosis-promoting effects of EnSC-EM-EC

Endometriotic lesions are surrounded by dense fibrous tissue; severe fibrosis may lead to pain, scarring and altered tissue functions, and it has a detrimental effect on the ovarian reserve and complicates surgical procedures [61]. However, the mechanisms of fibrogenesis in endometriotic lesions are poorly understood. In 2007, Yuge et al. demonstrated that compared with endometrial stromal cells, endometriotic stromal cells exhibit enhanced collagen gel contractility, myofibroblastic differentiation potential and fibronectin secretion [62]. Further study indicated that heparin can decrease the fibrogenic potential by suppressing Rho–ROCK-mediated pathway activation [63]. Accordingly, Li J et al. demonstrated that endometriotic MSCs significantly promote fibrogenesis in ovarian endometrioma through the Wnt/β-catenin pathway via paracrine production of TGF-β1 and Wnt1; additionally, treatment with CM from endometriotic MSCs markedly increased the mRNA expression levels of fibronectin and collagen I [64]. Consistent with the abovementioned reports, our results confirmed the overexpression of fibronectin in EnSC-EM-EC. As a multifunctional glycoprotein, fibronectin participates in cell adhesion, migration, phagocytosis and extracellular matrix formation, and the above findings collectively suggest that EnSC-EM-EC-secreted fibronectin may be involved in the progression of fibrogenesis in EMs [65].

### Potential signalling pathways involved in the dysfunction of EnSC-EM-EC

The unique characteristics of EnSC-EM-EC have been confirmed by an accumulating body of evidence; however, our observations are restricted to behavioural and functional studies. Indeed, several reports have indicated that the Toll-like receptor 4-mediated signalling pathway promotes the growth of endometriotic stromal cells and that the canonical Wnt/β-catenin pathway participates in fibrogenesis caused by endometriotic MSCs in ovarian endometrioma [64]. However, details of the potential signalling pathways underlying these abnormalities remain elusive. Therefore, in the present study, RNA-seq analysis was performed to identify the DEGs and potential signalling pathways responsible for the differences between EnSC-EM-EC and EnSC-Control. The 8 most relevant pathways were selected based on the DEGs and included the signalling pathway microRNAs in cancer, complement and coagulation cascades, cytokine-cytokine receptor interaction, ECM-receptor interaction, focal adhesion and ovarian steroidogenesis. The abovementioned dysregulated pathways are likely to be mediated through the canonical HIF-1, PI3K-Akt and TGF-β signalling pathways. Our results thus contribute further to the discovery of possible drug targets, because these genes and pathways are related to inhibiting the growth of endometriotic lesions by regulating EnSC-EM-EC activities, but have little or no off-target effects on the function of the normal endometrium.

### The origin of EnSC-EM-EC

Although EnSC-EM-EC and EnSC-Control exhibited morphological differences, the paracrine secretion of biological factors and the expression of adhesion molecules on both types of EnSCs were similar. Even among the more than 15,000 expressed genes, only 523 (3.487%) were significantly differentially expressed between EnSC-EM-EC and EnSC-Control. These findings strongly suggest that EnSC-EM-EC likely originate from EnSCs residing in the basal layer of the eutopic endometrium. Furthermore, EnSCs can be successfully isolated from menstrual blood, specifically from fragments of the shedding endometrium that are mixed with menstrual blood [20, 66]. This observation supports and promotes John Sampson’s implantation theory, which highlights the potential identity of EnSCs as the cells initiating ectopic lesions. Controversially, most women (75–90%) experience retrograde menstruation, but only approximately 10% of reproductive-aged women develop EMs, suggesting that additional mechanisms are involved [7, 13, 67].

Although the cloning efficiency of epithelial and stromal cells isolated from eutopic endometrium of EMs patients did not differ significantly, compared with that of cells from healthy eutopic endometrium [31], several recent studies have demonstrated differences in the genomic profile and stem cell marker expression between the eutopic endometrium of women with EMs and that of women without EMs [27–30, 68, 69]. Subsequently, Barragan et al. not only confirmed the genomic differences between endometrial MSCs isolated from eutopic endometrium of EMs patients or healthy controls but also indicated that endometrial MSCs are the progenitors of endometrial stromal fibroblasts, which inherit the P4-resistance phenotype from their endometrial MSC progenitors in EMs patients [70]. The above findings strongly suggest that dysfunction of eutopic EnSCs likely plays a critical role in promoting the formation of endometriotic lesions because endometrial regeneration is mainly accomplished by the proliferation and differentiation of eutopic EnSCs.

In addition, to our knowledge, the implantation theory could reasonably account for peritoneal and ovarian EMs but seems to be inadequate for explaining less common endometriotic lesions, such as those in the lung and brain (distal from the peritoneal cavity) [7, 13, 67]. However, Santamaria et al. demonstrated by fluorescent labelling in a mouse model that cells from endometriotic lesions can migrate to eutopic endometrium [71]. Subsequently, Cheng et al. also indicated that endometrial MSCs exhibit high tropism to endometriotic lesions, and a recent study further demonstrated that endometriotic MSCs, rather than differentiated cells from endometriotic lesions, can enter the vascular circulation, potentially through the CXCL12/CXCR4 axis, and lead to haematogenous dissemination [72]. The above findings not only demonstrate the interactions of EnSCs that reside in the eutopic endometrium and ectopic lesions of EMs patients but also provide a potential mechanism by which the less common endometriotic lesions distal to the peritoneal cavity are established.

Therefore, based on the published reports and our findings, we reasonably postulate that dysfunction of eutopic EnSCs plays a critical role in the onset of EMs, which directly causes the abnormal characteristics of eutopic endometrium. Subsequently, retrograde menstruation delivers deciduous abnormal endometrial tissues and EnSCs to the ovaries and pelvic cavity, and the survival of dysfunctional EnSCs is promoted via enhanced paracrine effects and the establishment of a favourable immune microenvironment. Endometriotic lesions are ultimately formed after continual recruitment of endometrial cells (including EnSCs) and a long latent period.

## Conclusion

Accumulating evidence has shed light on the importance of EnSCs in the establishment and progression of endometriotic lesions. The present study aimed to determine the differences between EnSC-EM-EC and EnSC-Control as well as identify the potential signalling pathways responsible for these abnormalities. Our results not only improve the understanding of EMs but also contribute to the use of EnSC-EM-EC as a therapeutic tool for drug discovery for EMs. The limitations of our study include the relatively small number of samples for analysis and the failure to collect eutopic endometrium from patients with EMs. Indeed, further studies to comprehensively explore the differences between EnSCs isolated from eutopic endometrium of individuals with and individuals without EMs would substantially promote the effective treatment and early diagnosis of EMs.

## Supplementary information

**Additional file 1: Figure S1.** Morphology of P3 EnSCs-Control and EnSC-EM-EC. EnSC-EM-EC isolated from independent patient (*n* = 5) were consistent and showed similar morphology, which display a more “aggressive” phenotype. **Figure S2.** Paracrine production of biological factors in the CM of EnSCs. The array images of EnSCs-Control (n = 5) and EnSC-EM-EC (*n* = 6) were shown. **Figure S3.** Expression of adhesion molecules on EnSCs. The array images of EnSCs-Control (n= 6) and EnSC-EM-EC (*n* = 7) were shown. **Figure S4.** GO functional classification on DEGs between EnSC-Control and EnSC-EM-EC.X axis means number of DEGs (the number is presented by its square root value); Y axis represents GO terms. All GO terms are grouped into three ontologies: blue is for biological process; brown is for cellular component and orange is for molecular function. **Figure S5.** KEGG classification on DEGs between EnSC-Control and EnSC-EM-EC. X axis means number of DEGs; Y axis represents second KEGG pathway terms. All second pathway terms are grouped in top pathway terms indicated in different color. METHODS. **Figure S6.** Identification of HUVECs. The HUVECs used in tube formation assay positively expressed typical endothelial markers, including CD31, VEGFR2 and vWF, and the positive ration exceeded 95%, which fulfill the standard of endothelial cells. **Figure S7.** The schematic diagram of CAM assay used in this study with minor improvement. the fertilized chicken eggs were incubated at 38.2°C with approximately 55-65% humidity under sterile conditions. On day 3, the shallow notch was made on the shell with saw blade, and 3 to 5 ml of albumen were removed by sterilized syringe to allow detachment of the developing CAM from the shell. Subsequently, the small hole was sealed with tape, and the eggs were returned to the incubator with the fixed position. On day 7, an opening window was made by scissor on the shell, and a sterilized silicone loop with diameter of 10 mm was placed on top of the growing CAM between mature blood vessels. **Table S1.** Details of antibodies used. **Table S2.** The DEGs between EnSC-Control and EnSC-EM-EC. **Table S3.** The well-chosen top 8 pathway enrichment of DEGs between EnSC-Control and EnSC-EM-EC.

## Data Availability

The datasets used and/or analysed during the current study are available from the corresponding author on reasonable request.

## Abbreviations

EMs: Endometriosis
EnSCs: Endometrial stem cells
EnSC-EM-EC: EnSCs isolated from ectopic lesions of EMs patients
EnSC-Control: EnSCs isolated from eutopic endometrium of control individuals
MSC: Mesenchymal stem cells
DEGs: Differentially expressed genes
CM: Conditioned medium
