# Supplementary material for: Biological characteristics of endometriotic mesenchymal stem cells isolated from ectopic lesions of patients with endometriosis
Source: Stem Cell Res Ther. 2020 Aug 8;11:346. doi: 10.1186/s13287-020-01856-8 (PMC7414689; doi:10.1186/s13287-020-01856-8)
Supplement: Supplementary file 1 — Additional file 1: Figure S1. Morphology of P3 EnSCs-Control and EnSC-EM-EC. EnSC-EM-EC isolated from independent patient (n = 5) were consistent and showed similar morphology, which display a more “aggressive” phenotype. Figure S2. Paracrine production of biological factors in the CM of EnSCs. The array images of EnSCs-Control (n = 5) and EnSC-EM-EC (n = 6) were shown. Figure S3. Expression of adhesion molecules on EnSCs. The array images of EnSCs-Control (n= 6) and EnSC-EM-EC (n = 7) were shown. Figure S4. GO functional classification on DEGs between EnSC-Control and EnSC-EM-EC.X axis means number of DEGs (the number is presented by its square root value); Y axis represents GO terms. All GO terms are grouped into three ontologies: blue is for biological process; brown is for cellular component and orange is for molecular function. Figure S5. KEGG classification on DEGs between EnSC-Control and EnSC-EM-EC. X axis means number of DEGs; Y axis represents second KEGG pathway terms. All second pathway terms are grouped in top pathway terms indicated in different color. METHODS. Figure S6. Identification of HUVECs. The HUVECs used in tube formation assay positively expressed typical endothelial markers, including CD31, VEGFR2 and vWF, and the positive ration exceeded 95%, which fulfill the standard of endothelial cells. Figure S7. The schematic diagram of CAM assay used in this study with minor improvement. the fertilized chicken eggs were incubated at 38.2°C with approximately 55-65% humidity under sterile conditions. On day 3, the shallow notch was made on the shell with saw blade, and 3 to 5 ml of albumen were removed by sterilized syringe to allow detachment of the developing CAM from the shell. Subsequently, the small hole was sealed with tape, and the eggs were returned to the incubator with the fixed position. On day 7, an opening window was made by scissor on the shell, and a sterilized silicone loop with diameter of 10 mm was placed on top of the growing CAM be [file 13287_2020_1856_MOESM1_ESM.pdf]

Supplemental Information

Biological characteristics of endometriotic mesenchymal stem cells isolated from ectopic lesions of patients with endometriosis

Yanli Liu, Shengying Liang, Fen Yang, Lidan Niu, Yuliang Sun, Yakun Ren, Hongmei Wang, Yanan He, Jiang Du, Xinxing Zhu, Jun Yang, Juntang Lin

Supplemental Figure S1. Liu et al.

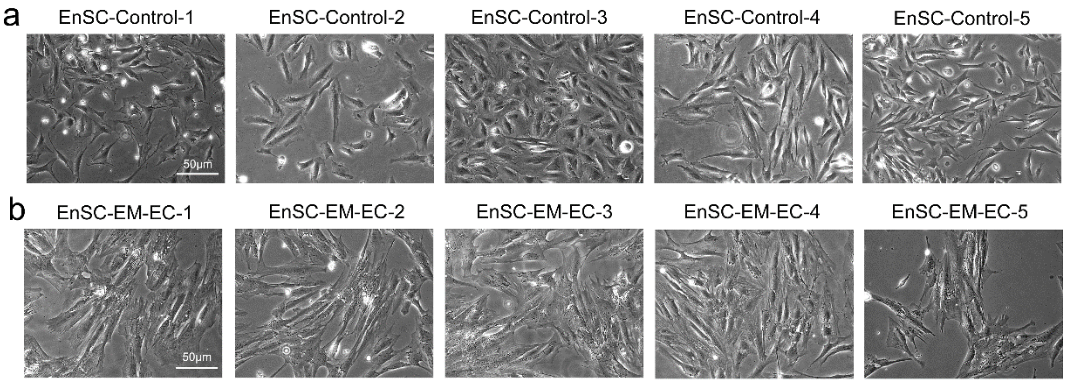

Figure S1. Morphology of P3 EnSCs-Control and EnSC-EM-EC. EnSC-EM-EC isolated from independent patient (n = 5) were consistent and showed similar morphology, which display a more “aggressive” phenotype.

Supplemental Figure S2. Liu et al.

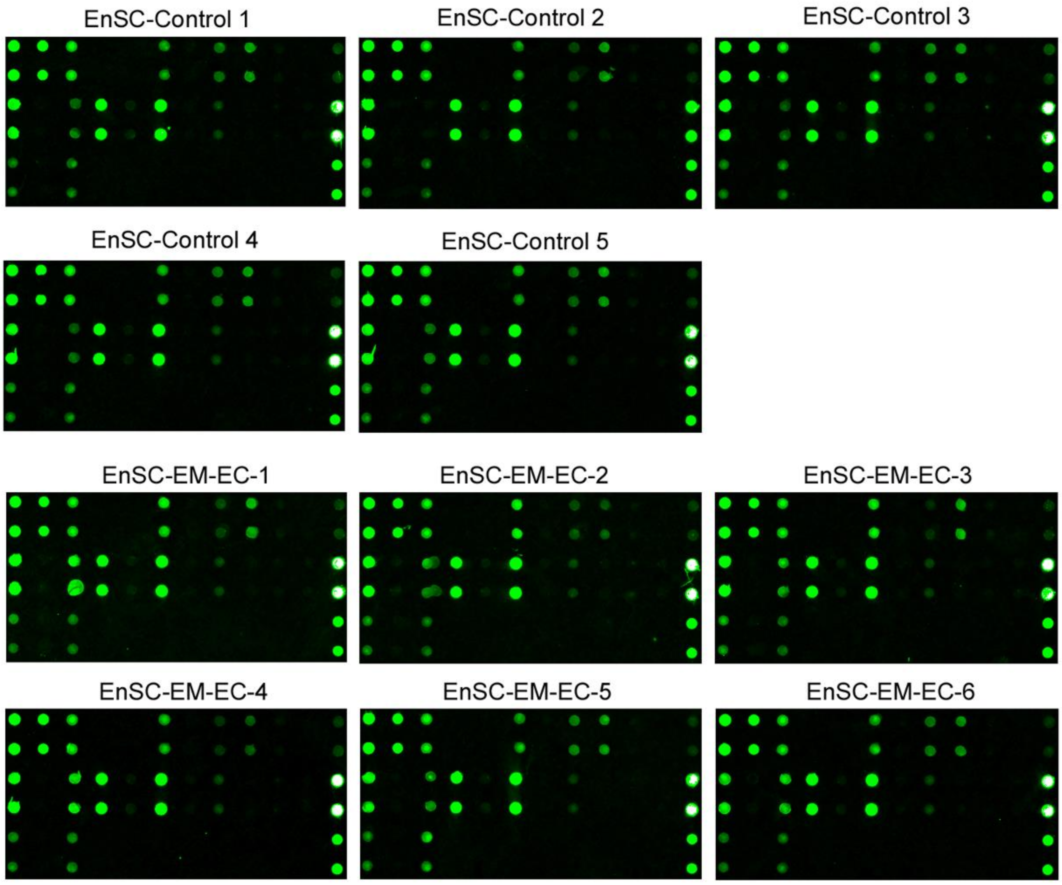

Figure S2. Paracrine production of biological factors in the CM of EnSCs. The array images of EnSCs-Control (n = 5) and EnSC-EM-EC (n = 6) were shown.

Supplemental Figure S3. Liu et al.

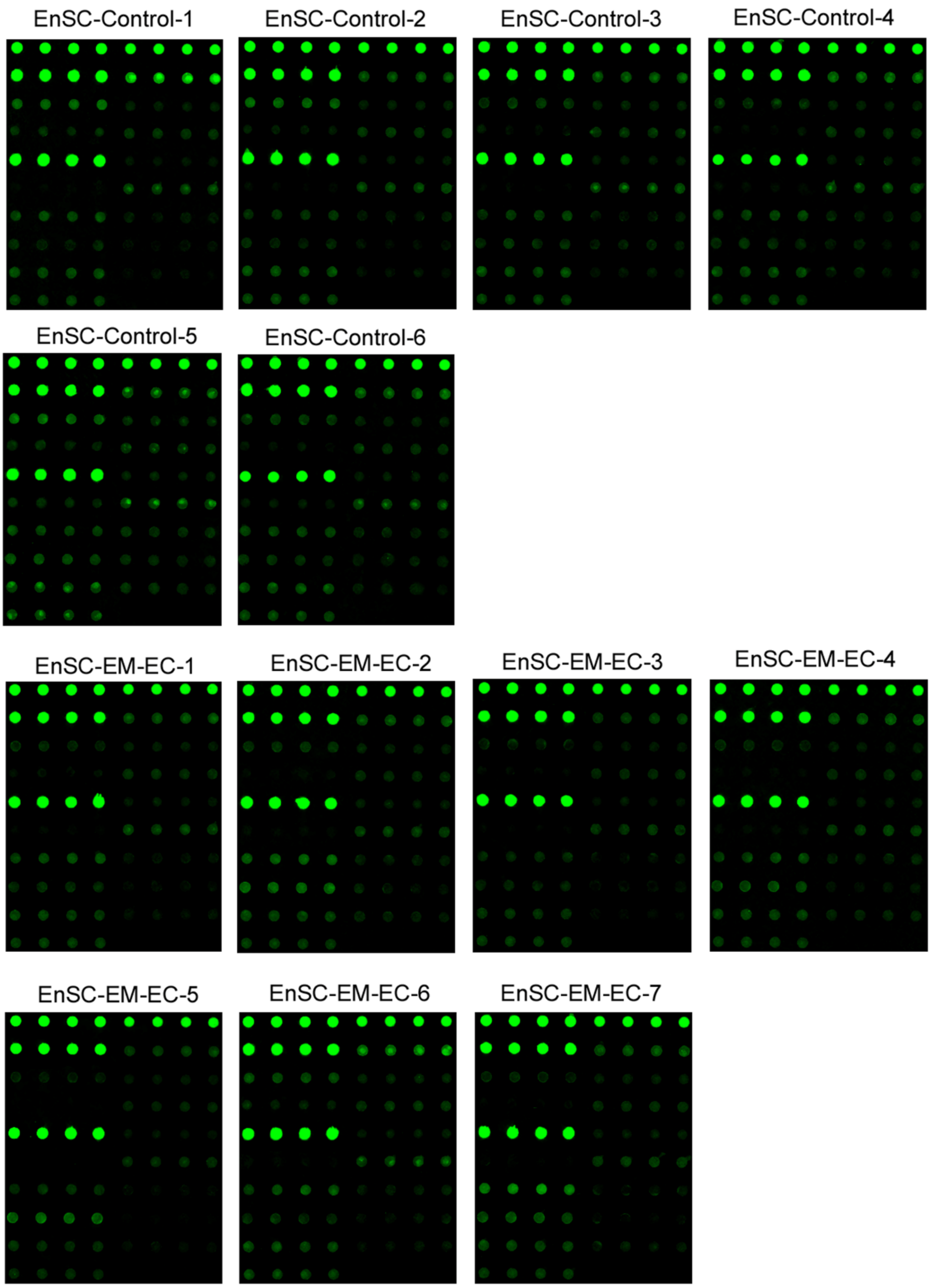

Figure S3. Expression of adhesion molecules on EnSCs. The array images of EnSCs-Control (n = 6) and EnSC-EM-EC (n = 7) were shown.

Supplemental Figure S4. Liu et al.

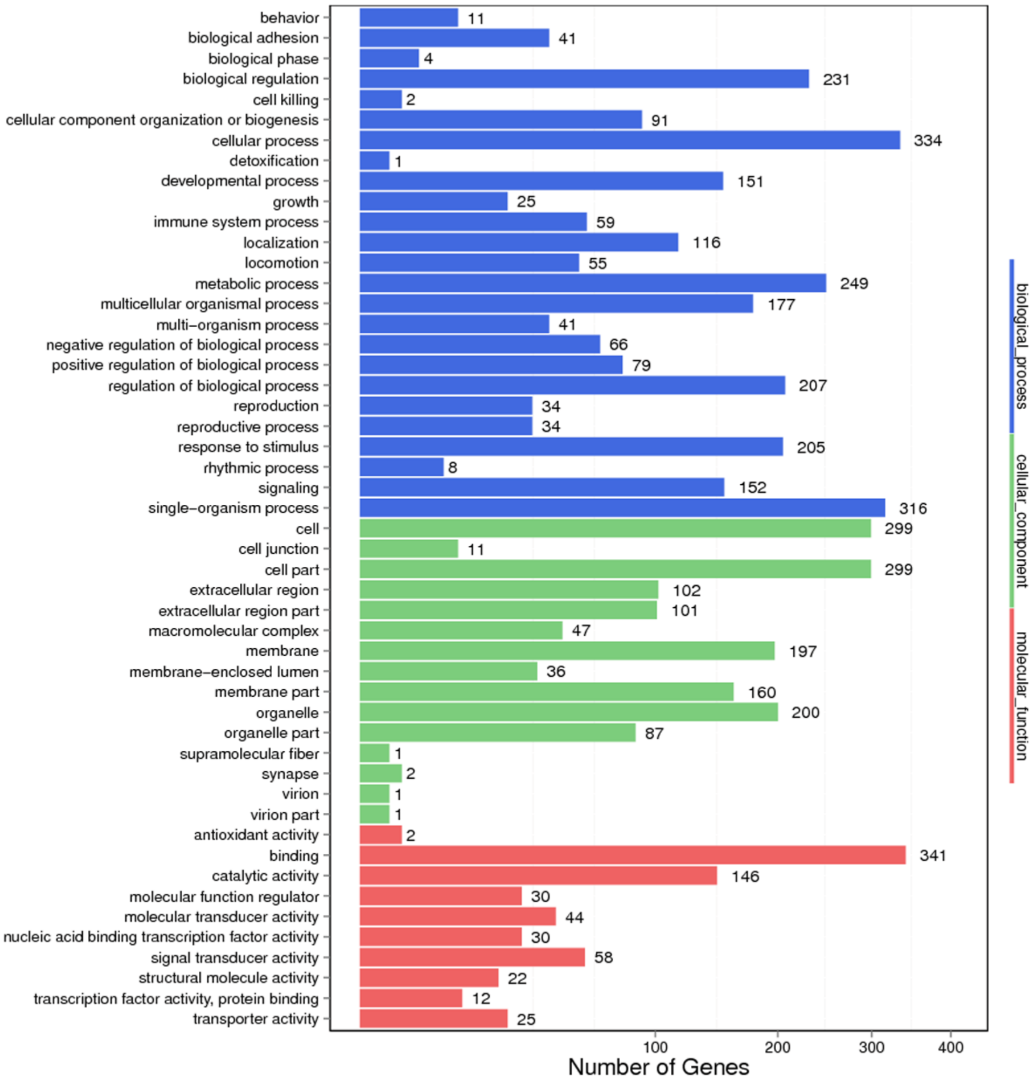

Figure S4. GO functional classification on DEGs between EnSC-Control and EnSC-EM-EC. X axis means number of DEGs (the number is presented by its square root value); Y axis represents GO terms. All GO terms are grouped into three ontologies: blue is for biological process; brown is for cellular component and orange is for molecular function.

Supplemental Figure S5. Liu et al.

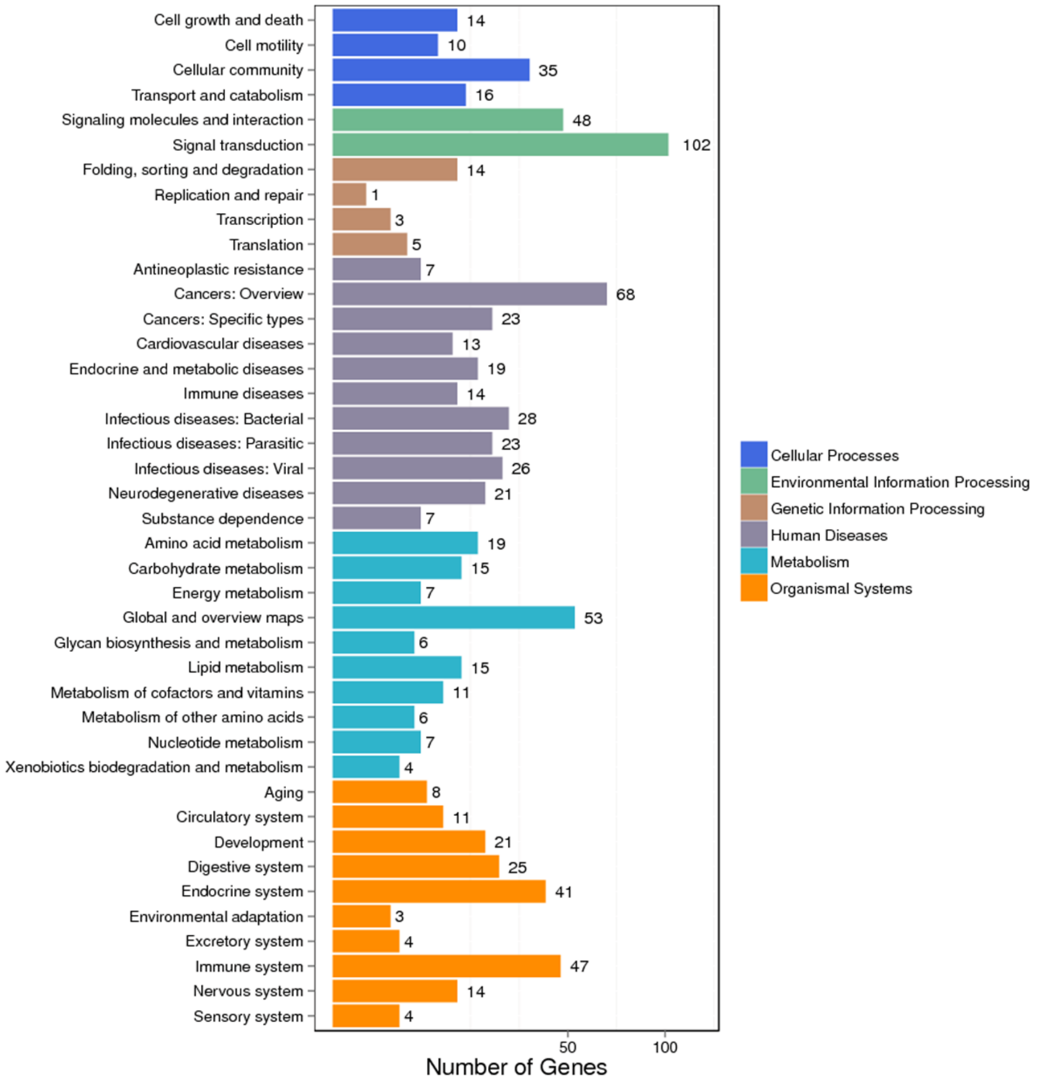

Figure S5. KEGG classification on DEGs between EnSC-Control and EnSC-EM-EC. X axis means number of DEGs; Y axis represents second KEGG pathway terms. All second pathway terms are grouped in top pathway terms indicated in different color.

METHODS

Isolation and Culture of human umbilical cord venous endothelial cells (HUVECs). HUVECs isolation was performed as described before. Briefly, human umbilical cord (20 cm) was rinsed by PBS with P/S in sterile conditions, tidily cut both ends of the cord with a scalpel, cannulated at both ends with sterile needles and tightly maintain them with string. Subsequently, PBS with P/S was introduced into umbilical vein through the needles fixed at the end of umbilical cord, in order to completely wash the red blood cells and blood clots away. Next, after incubation with 0.2% collagenase solution (Sigma-Aldrich) in 37℃ water-bath for 10 min, the umbilical vein was gently squeezed to facilitate cell detachment. Then, the umbilical vein was fulfilled with complete ECM medium, and the cell suspension was collected into a sterile tube, centrifuged for 10 min at 750 g. Finally, the pellet of cells were gently resuspended in complete ECM medium and seeded into cell culture flasks previously treated with fibronectin solution (Sigma-Aldrich) at 37℃ with 5% humidified CO_2. After reaching confluence (P0), the cells were detached by 0.25% trypsin/1 mM EDTA and subcultured to new flasks by the ratio of 1: 3, and P3 HUVECs were used for the further tests.

Supplemental Figure S6. Liu et al.

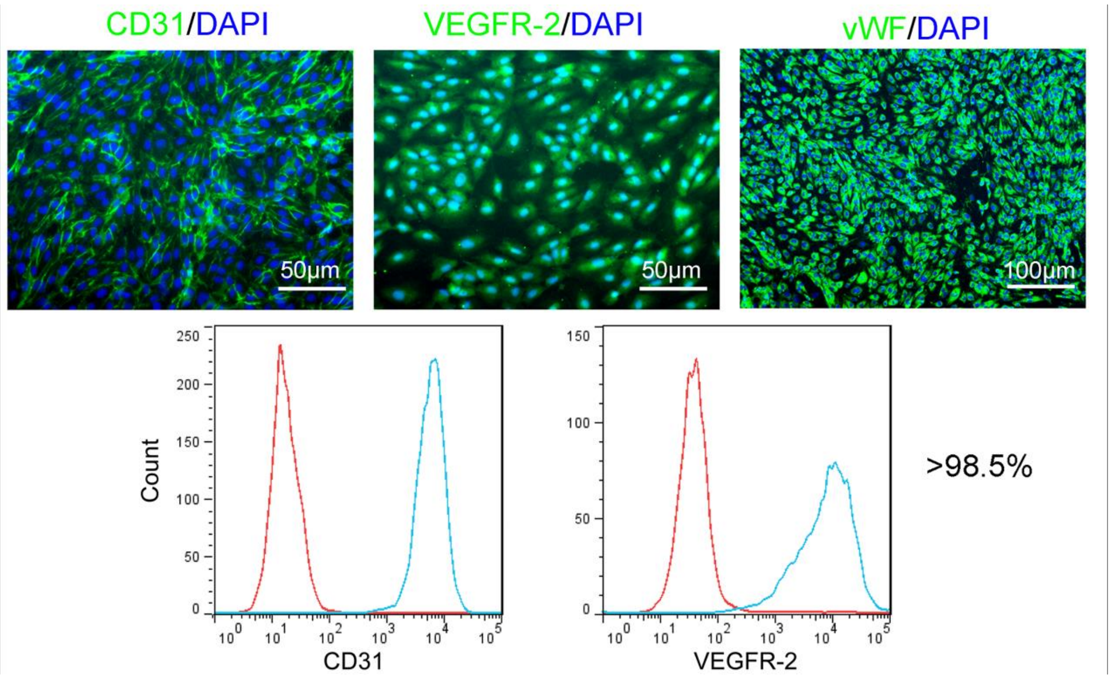

Fig S6. Identification of HUVECs. The HUVECs used in tube formation assay positively expressed typical endothelial markers, including CD31, VEGFR2 and vWF, and the positive ration exceeded 95%, which fulfill the standard of endothelial cells.

Supplemental Figure S7. Liu et al.

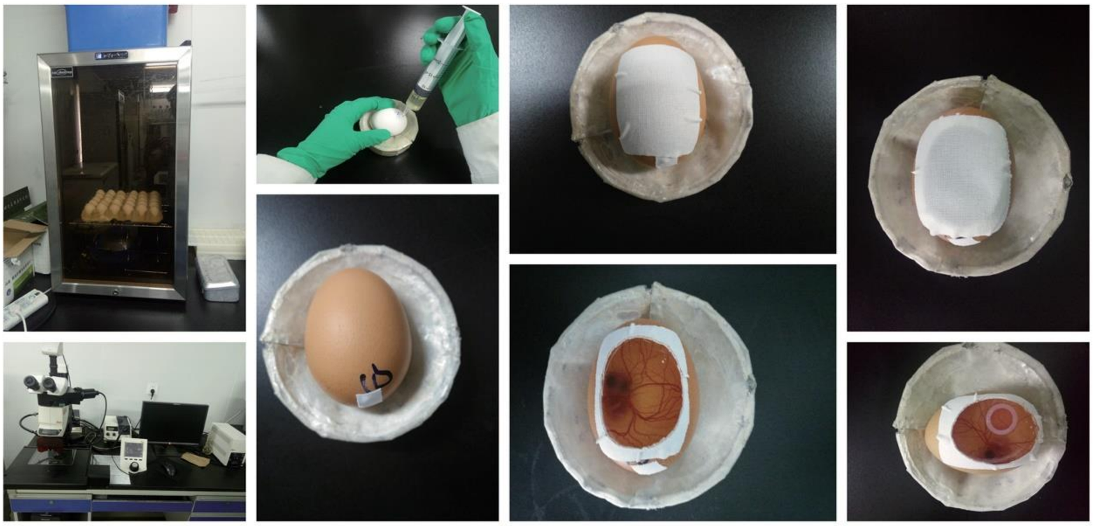

Fig S7 The schematic diagram of CAM assay used in this study with minor improvement. the fertilized chicken eggs were incubated at 38.2 ℃ with approximately 55-65% humidity under sterile conditions. On day 3, the shallow notch was made on the shell with saw blade, and 3 to 5 ml of albumen were removed by sterilized syringe to allow detachment of the developing CAM from the shell. Subsequently, the small hole was sealed with tape, and the eggs were returned to the incubator with the fixed position. On day 7, an opening window was made by scissor on the shell, and a sterilized silicone loop with diameter of 10 mm was placed on top of the growing CAM between mature blood vessels.

Table S1 Details of antibodies used

Antibodies	Manufacturer	Catalog No	Test	Concentration used
Anti-human monoclonal rabbit Vimentin	Abcam	ab92547	IF	1: 300
FITC conjugated anti-human monoclonal mouse CD29	eBioscience	11-0299-42	FC	1: 20
FITC conjugated anti-human monoclonal mouse CD73	eBioscience	11-0739-41	FC	1: 20
FITC conjugated anti-human monoclonal mouse CD90	eBioscience	11-0909-41	FC	1: 20
PE conjugated anti-human monoclonal mouse CD105	eBioscience	12-1057-41	FC	1: 20
FITC conjugated anti-human monoclonal mouse CD34	eBioscience	11-0349-42	FC	1: 20
FITC conjugated anti-human monoclonal mouse CD45	eBioscience	11-9459-42	FC	1: 20
FITC conjugated anti-human monoclonal mouse HLA-ABC	eBioscience	11-9983-42	FC	1: 20
FITC conjugated anti-human monoclonal mouse HLA-DR	eBioscience	11-9956-42	FC	1: 20
FITC/PE conjugated monoclonal mouse IgG1/IgG2a isotype control cocktail	Invitrogen	PA5-33181	FC	1: 20
Anti-human monoclonal mouse CD31	Abcam	ab9498	IF/FC	1: 300 for IF; 1: 100 for FC
Anti-human monoclonal mouse VEGFR2	Abcam	ab9530	IF/FC	1: 300 for IF; 1: 100 for FC
Anti-human polyclonal rabbit vWF	Abcam	ab6994	IF	1: 300
Anti-human polyclonal rabbit Fibronectin	Abcam	ab2413	IF	1: 300
Alexa Fluor 488 conjugated goat anti-rabbit IgG secondary antibody,	Invitrogen	A27034	IF	1:1000
Alexa Fluor 488 conjugated goat anti-mouse IgG secondary antibody	Invitrogen	A28175	IF/FC	1:1000 for IF; 1: 250 for FC
GAPDH (D16H11) XP® Rabbit mAb	CST	#5174	WB	1:1000
Akt (pan) (C67E7) Rabbit mAb	CST	#4691	WB	1:1000

Phospho-Akt (Ser473) (D9E) XP® Rabbit mAb	CST	#4060	WB	1:1000
SAPK/JNK Antibody	CST	#9252	WB	1:1000
Phospho-p44/42 MAPK (Erk1/2) (Thr202/Tyr204) (D13.14.4E) XP® Rabbit mAb	CST	#4370	WB	1:1000
HRP Conjugate Goat Anti-Rabbit IgG (H+L), Secondary Antibody	BOSTER	BA1055	WB	1:5000

Table S2 The DEGs between EnSC-Control and EnSC-EM-EC

GeneID	geneLength	Means-EnSC-Control	Means-EnSC-EM-EC	log2Ratio(EnSC-EM-EC/EnSC-Control)	Up-Down-Regulation(EnSC-EM-EC/EnSC-Control)	Probability	Symbol
4879	708	4.166	887.1166667	7.734317199	Up	0.997999768	NPPB
5740	5603	2.22	224.9166667	6.662687084	Up	0.992372665	PTGIS
2202	3060.04	2.876	223.9666667	6.283076544	Up	0.991214758	EFEMP1
6424	2974	0.406	84.345	7.69867901	Up	0.985369532	SFRP4
4319	1777	375.78	13.81333333	-4.765754877	Down	0.982749739	MMP10
4314	1906	1474.346	60.70666667	-4.602076367	Down	0.982701009	MMP3
4345	2318.45	1.422	82.12833333	5.851886651	Up	0.980071934	CD200
27295	2709.57	0.244	58.54	7.906397788	Up	0.979828286	PDLIM3
9220	2110	52.6	0.153333333	-8.422247629	Down	0.978043857	TIAF1
8828	5686.93	6.318	155.9666667	4.625625981	Up	0.976059868	NRP2
5270	2256.44	34.126	626.3483333	4.198021984	Up	0.974985497	SERPINE2
7045	2805	93.134	1656.055	4.152298835	Up	0.974502843	TGFBI
4256	1398	339.746	18.53333333	-4.196262278	Down	0.973567699	MGP
1469	782	68.946	2.15	-5.003058289	Down	0.971251885	CST1
8076	2945.51	1.62	59.32	5.19445288	Up	0.970288897	MFAP5
23231	4411.84	1.074	49.25666667	5.519253102	Up	0.969121708	SEL1L3
28984	1126	208.686	12.88833333	-4.017196201	Down	0.967518274	RGCC
27129	2908	5.474	105.9666667	4.274871271	Up	0.966738601	HSPB7
56925	1132	6.6	118.3916667	4.164957702	Up	0.965176935	LXN
2627	3785	0.612	37.29	5.929113334	Up	0.964053835	GATA6
5806	1955	0.544	35.69166667	6.03583681	Up	0.963246316	PTX3
6275	523.13	217.912	15.84666667	-3.781494337	Down	0.961566307	S100A4
1589	2041	58.994	2.835	-4.379147592	Down	0.959512704	CYP21A2
11346	5390.18	6.71	100.9383333	3.911017594	Up	0.959085741	SYNPO
2335	8529.21	248.834	2728.853333	3.455039327	Up	0.958874579	FN1
5972	1493	60.876	3.385	-4.168645827	Down	0.957032138	REN
3481	4593.25	479.276	45.04666667	-3.411364439	Down	0.954064277	IGF2

56971	2262	60.974	4.02	-3.922926786	Down	0.951572108	CEACAM19
158471	8318.15	0.35	25.77666667	6.20256698	Up	0.951551224	PRUNE2
54541	1752	7.502	93.42333333	3.638435753	Up	0.950701938	DDIT4
58189	1472	30.948	0.99	-4.966273844	Down	0.95056271	WFDC1
5142	3884.02	41.316	2.113333333	-4.289108342	Down	0.949801601	PDE4B
84293	1713.26	113.876	9.86	-3.529732266	Down	0.949683258	FAM213A
4015	5330	67.224	627.24	3.221969271	Up	0.948717949	LOX
205	6890.42	0.482	24.87666667	5.689616229	Up	0.947367444	AK4
2167	838	21.966	0.165	-7.056662345	Down	0.946346444	FABP4
1803	3913	2.84	41.96166667	3.885109146	Up	0.941053486	DPP4
90865	2561.33	0.064	19.39666667	8.243521125	Up	0.940828402	IL33
10763	5591	45.552	3.441666667	-3.726335117	Down	0.940215802	NES
7148	8349.36	115.106	12.19666667	-3.238404217	Down	0.939979116	TNXB
25890	4488	1.09	26.14166667	4.583951083	Up	0.939009166	ABI3BP
6281	1069	29.962	243.9866667	3.025596476	Up	0.938869939	S100A10
768	1561	0.206	19.18666667	6.541315944	Up	0.937419654	CA9
9518	1417	150.764	17.30833333	-3.122753262	Down	0.937338438	GDF15
5010	2742.81	16.562	142.5383333	3.105401155	Up	0.936612136	CLDN11
72	1344.28	59.948	465.035	2.955555774	Up	0.93657965	ACTG2
9358	2620.41	1.22	26.395	4.435311613	Up	0.936500754	ITGBL1
1809	5208.96	20.528	166.1566667	3.016879198	Up	0.936036663	DPYSL3
4502	466	185	1345.931667	2.86300799	Up	0.935554009	MT2A
684	1048	5.86	58.815	3.327211573	Up	0.934454113	BST2
10417	1885.67	531.352	75.235	-2.820192029	Down	0.933428472	SPON2
9244	1824	0.132	17.53833333	7.053829915	Up	0.933126813	CRLF1
1734	6148	23.308	0.995	-4.54998488	Down	0.932565263	DIO2
56901	1275	0.712	21.095	4.888880036	Up	0.931869126	NDUFA4L2
3485	1234.25	20.672	162.4983333	2.974675041	Up	0.931456085	IGFBP2
114769	753.95	90.34	10.82	-3.061664415	Down	0.930616081	CARD16
140862	2610	17.02	0.148333333	-6.842244391	Down	0.930592876	ISM1
639	4753	47.46	4.536666667	-3.387007534	Down	0.930553428	PRDM1
4316	1153	19.176	0.508333333	-5.237383167	Down	0.92989906	MMP7

1311	2471	0.03	15.99833333	9.0587434	Up	0.929077619	COMP
3207	2653	44.116	4.418333333	-3.319727818	Down	0.927481146	HOXA11
10267	920	53.296	5.755	-3.211139426	Down	0.927418494	RAMP1
6035	833.66	0.032	15.46	8.916252699	Up	0.926578489	RNASE1
1009	6819.63	13.33	102.9683333	2.949452037	Up	0.92653208	CDH11
2331	3271	5.2	49.50833333	3.251087854	Up	0.926293073	FMOD
151887	4674.93	47.74	322.27	2.754999423	Up	0.926211858	CCDC80
1294	9169	46.41	4.775	-3.280863059	Down	0.925348648	COL7A1
84624	6578	0.32	16.8	5.714245518	Up	0.925130526	FNDC1
2626	3130.47	0.142	15.345	6.755733906	Up	0.923327532	GATA4
2624	3263	52.192	5.936666667	-3.136103671	Down	0.922561782	GATA2
91461	2517	1.248	22.13833333	4.148856775	Up	0.922148741	PKDCC
144347	2160	0.6	17.92333333	4.90073266	Up	0.922113934	RFLNA
5328	2398	182.29	27.65833333	-2.720449292	Down	0.921849402	PLAU
80303	1868.75	30.778	2.69	-3.516221408	Down	0.921352825	EFHD1
2516	3095	0.01	14.19833333	10.47150587	Up	0.920607959	NR5A1
6352	1237	79.84	11.16166667	-2.838559253	Down	0.92015083	CCL5
5352	4071.38	26.21	168.345	2.683231614	Up	0.91984453	PLOD2
1301	7290.95	0.206	15.01	6.187135829	Up	0.919800441	COL11A1
9351	2092.22	150.14	23.03333333	-2.704513273	Down	0.919721545	SLC9A3R2
5999	3223.7	6.456	51.91833333	3.007531587	Up	0.919561434	RGS4
1439	4848	62.384	8.021666667	-2.959202137	Down	0.919125189	CSF2RB
2621	2551	35.48	214.9616667	2.599001497	Up	0.919078779	GAS6
123	2029	34.808	209.6066667	2.590193776	Up	0.918670379	PLIN2
51339	3798.75	1.288	21.49833333	4.06102032	Up	0.918505627	DACT1
1E+08	3887	142.392	22.29	-2.675399572	Down	0.918410488	HOTS
3223	1869.04	0.688	17.62333333	4.678934451	Up	0.918294466	HOXC6
6387	2221.38	66.816	9.103333333	-2.875726804	Down	0.918194686	CXCL12
4312	1903	108.228	16.31666667	-2.729655527	Down	0.917234018	MMP1
4320	2306	46.398	5.695	-3.026294872	Down	0.917185288	MMP11
59	1418.99	441.24	2530.181667	2.519605486	Up	0.916493793	ACTA2
5318	4390.25	0.294	14.75	5.648754989	Up	0.915570252	PKP2

644150	4195	2.088	25.21833333	3.594279315	Up	0.915486715	WIPF3
10840	3003	14.294	0.211666667	-6.077471792	Down	0.915444947	ALDH1L1
1674	2268	7.674	56.75666667	2.886739192	Up	0.915403179	DES
400916	718	107.36	16.85666667	-2.671065394	Down	0.915157211	CHCHD10
7052	5026.22	442.378	76.58166667	-2.530208677	Down	0.914922845	TGM2
6347	760	20.42	124.3333333	2.606158358	Up	0.914911243	CCL2
79627	8875.22	97.672	15.19833333	-2.684031914	Down	0.914358974	OGFRL1
347735	2428.97	86.98	13.59333333	-2.677784434	Down	0.912716092	SERINC2
1475	838	0.158	13.54833333	6.422047019	Up	0.912611672	CSTA
3294	1451	13.702	0.2	-6.09824268	Down	0.912147581	HSD17B2
7378	1503.58	31.286	3.315	-3.238436442	Down	0.911961945	UPP1
7076	931	480.896	2544.731667	2.403716706	Up	0.911760065	TIMP1
216	2378	71.872	10.845	-2.72839978	Down	0.911384151	ALDH1A1
7022	2895	27.174	2.628333333	-3.370006786	Down	0.911235642	TFAP2C
57580	6633	36.102	4.295	-3.071348726	Down	0.910590556	PREX1
84561	3449	2.506	26.43833333	3.399172913	Up	0.910548788	SLC12A8
5669	1985	44.938	5.938333333	-2.919805935	Down	0.910360831	PSG1
5648	3893.12	82.686	13.54833333	-2.609527692	Down	0.909780717	MASP1
1E+08	2401.25	70.874	10.98	-2.69037842	Down	0.909741269	CD24
4232	2442.71	227.05	40.855	-2.47442548	Down	0.909683258	MEST
4803	1052	0.472	14.70666667	4.96153962	Up	0.909277178	NGF
7869	2916.78	58.126	8.461666667	-2.780169872	Down	0.909096183	SEMA3B
11142	1199.27	259.312	48.11666667	-2.430080363	Down	0.908903585	PKIG
7058	5898	28.874	164.1166667	2.50687887	Up	0.908850215	THBS2
92949	7858	0.134	12.85166667	6.583578656	Up	0.908748115	ADAMTSL1
3885	1713	69.046	11.05166667	-2.643293884	Down	0.907750319	KRT34
9510	4670	207.772	38.58	-2.429076197	Down	0.907529876	ADAMTS1
54757	4305.1	1.052	17.85166667	4.084852164	Up	0.907404571	FAM20A
64866	6027	6.182	43.93166667	2.829115674	Up	0.907274626	CDCP1
2151	3446	42.402	5.845	-2.858857385	Down	0.907086669	F2RL2
4493	519	11.774	70.81	2.588348584	Up	0.906622578	MT1E
23114	9767.03	1.664	20.82666667	3.645704614	Up	0.906246664	NFASC

176	8840	0.286	13.15166667	5.523086682	Up	0.905395057	ACAN
5350	3021	1.316	18.74833333	3.832530956	Up	0.905292957	PLN
6376	3217	13.162	0.291666667	-5.495914401	Down	0.905211742	CX3CL1
5798	3634.15	1.862	21.685	3.541772465	Up	0.905067873	PTPRN
794	1469.92	0.962	16.67666667	4.115650247	Up	0.904401903	CALB2
6372	1677	0.37	13.46166667	5.185187958	Up	0.904353173	CXCL6
11096	9680	31.228	3.838333333	-3.024288264	Down	0.904260355	ADAMTS5
3732	1667.05	86.504	14.65833333	-2.561045768	Down	0.903603666	CD82
335	1035.7	0.11	12.03	6.772989309	Up	0.903510848	APOA1
79887	1951	28.894	3.385	-3.093542201	Down	0.902615153	PLBD1
1152	1475	56.586	9.431666667	-2.584860522	Down	0.902190509	CKB
3236	1814	27.992	3.198333333	-3.129622359	Down	0.902123216	HOXD10
2537	837.14	105.76	19.47166667	-2.441345801	Down	0.901721777	IFI6
4052	5647.68	3.08	27.31	3.148427058	Up	0.901441002	LTBP1
1545	5160	8.826	53.66666667	2.60419463	Up	0.901278571	CYP1B1
629	2646	1.162	17.33833333	3.899283251	Up	0.900904977	CFB
2199	4200.11	12.062	71.15833333	2.56056358	Up	0.900436246	FBLN2
3237	1463	28.35	3.468333333	-3.031034271	Down	0.90040608	HOXD11
3037	3275	1.542	18.98166667	3.621732002	Up	0.90030398	HAS2
664	1661	27.362	137.4233333	2.328381391	Up	0.900243648	BNIP3
10085	4751.81	13.064	75.09	2.523024096	Up	0.900025525	EDIL3
64968	1017	115.654	22.33	-2.372760007	Down	0.89994895	MRPS6
3486	2620.11	189.07	906.5183333	2.26141573	Up	0.8986727	IGFBP3
8622	4345	15.776	0.958333333	-4.041060096	Down	0.898556677	PDE8B
4784	5570.45	17.554	92.65	2.399991164	Up	0.898364079	NFIX
6423	2005	0.052	11.03	7.728705452	Up	0.897677225	SFRP2
9537	3345.26	56.282	279.2933333	2.311035636	Up	0.897644738	TP53I11
2825	2168.01	1.384	17.88333333	3.691699822	Up	0.897370925	GPR1
283208	2299	1.028	15.95166667	3.955794998	Up	0.897333797	P4HA3
5155	2997.06	29.894	3.883333333	-2.944488497	Down	0.897201532	PDGFB
5236	2483.26	15.778	83.65666667	2.406566172	Up	0.897115675	PGM1
64220	2752.81	25.634	3.011666667	-3.089424613	Down	0.897029818	STRA6

56265	2409	38.834	5.911666667	-2.715683487	Down	0.896521638	CPXM1
23475	2174.64	35.996	5.226666667	-2.783873539	Down	0.895848706	QPRT
7993	1437.5	3.838	29.14166667	2.924656774	Up	0.895540086	UBXN8
3224	2290	0.546	13.16	4.591114728	Up	0.895419422	HOXC8
10082	7114	1.398	17.40333333	3.637927392	Up	0.894939088	GPC6
1634	4270.08	10.754	59.695	2.472736721	Up	0.893899524	DCN
51477	2351.09	68.174	12.855	-2.406892023	Down	0.893751015	ISYNA1
5918	1459.74	0.596	13.20666667	4.469810239	Up	0.893614108	RARRES1
220	3622	6.348	39.11666667	2.623409403	Up	0.893303167	ALDH1A3
23245	3344.8	56.386	10.08166667	-2.483602842	Down	0.892936536	ASTN2
5054	3207	449.416	1929.221667	2.101895525	Up	0.890804038	SERPINE1
63898	3272.81	4.43	30.82333333	2.798644284	Up	0.890474533	SH2D4A
56892	1841	25.336	3.261666667	-2.957507517	Down	0.890212322	C8orf4
7436	5230	3.464	26.05833333	2.911233978	Up	0.890070774	VLDLR
10157	5887	23.168	2.898333333	-2.998838076	Down	0.889906022	AASS
643314	6979	3.664	26.68833333	2.864717805	Up	0.889736628	KIAA0754
5307	2383	21.57	2.431666667	-3.149008794	Down	0.889274858	PITX1
57493	9156	51.798	232.085	2.163684983	Up	0.889103144	HEG1
3280	1475	107.472	22.13666667	-2.279450936	Down	0.888636733	HES1
3162	1606	8.29	46.665	2.492896889	Up	0.888478942	HMOX1
55194	1004.21	169.972	37.8	-2.168838967	Down	0.887780485	EVA1B
6770	2695	2.366	20.91166667	3.143786071	Up	0.887402251	STAR
1906	2112	21.302	2.498333333	-3.091951006	Down	0.88729551	EDN1
2114	3784	50.61	9.53	-2.408874355	Down	0.887253742	ETS2
5230	2439	84.09	354.4033333	2.075386023	Up	0.886942801	PGK1
3075	3976.6	2.726	21.81833333	3.000683434	Up	0.886506555	CFH
57451	9501.52	0.14	10.23	6.191235508	Up	0.886404455	TENM2
2308	5738	28.356	4.206666667	-2.752904626	Down	0.885694396	FOXO1
4811	5903	361.676	87.20666667	-2.052187534	Down	0.885350969	NID1
5032	1977	9.566	0.01	-9.901771981	Down	0.884993619	P2RY11
11343	4307.94	164.282	38.08666667	-2.108816482	Down	0.883849635	MGLL
4920	4099	0.568	11.97	4.397388412	Up	0.883687203	ROR2

718	5148	23.688	106.9716667	2.175000423	Up	0.883550296	C3
9435	4154	16.47	1.555	-3.40485407	Down	0.882930734	CHST2
6586	9727	1.146	14.67166667	3.678353818	Up	0.882891287	SLIT3
57124	2576	353.436	88.14333333	-2.003525635	Down	0.882814712	CD248
3357	2027.67	40.012	7.341666667	-2.446253225	Down	0.882290289	HTR2B
3908	9708	0.534	11.61166667	4.44259151	Up	0.881299455	LAMA2
27242	3646	76.164	16.255	-2.228225692	Down	0.88117415	TNFRSF21
5671	1922	11.164	0.46	-4.601076358	Down	0.880853927	PSG3
84206	3534	2.654	20.9	2.977262666	Up	0.880213482	MEX3B
668	2917	86.392	19.26333333	-2.165040354	Down	0.880180995	FOXL2
116496	6969	6.53	36.22333333	2.471764415	Up	0.87989558	FAM129A
220108	4808	35.296	6.283333333	-2.489902673	Down	0.879751711	FAM124A
2159	1558.92	35.938	6.501666667	-2.466628625	Down	0.879600882	F10
1612	5824.98	0.52	11.35666667	4.448884013	Up	0.879313145	DAPK1
4629	6882	1.796	17.02	3.244371782	Up	0.879269057	MYH11
2118	2242.14	25.504	3.91	-2.705483022	Down	0.879194802	ETV4
219	3088	24.002	103.6266667	2.110168775	Up	0.879071818	ALDH1B1
8436	3265	3.426	23.80666667	2.796768577	Up	0.878396566	SDPR
23266	5989.48	2.074	18.16	3.130276403	Up	0.878238775	ADGRL2
8869	2354.73	38.866	7.623333333	-2.350014771	Down	0.877178327	ST3GAL5
2026	2423	12.298	58.4	2.247544657	Up	0.877171366	ENO2
10570	2732	1.558	15.54666667	3.318838149	Up	0.877159763	DPYSL4
55504	4214	54.884	11.33333333	-2.275813385	Down	0.877138879	TNFRSF19
1075	2566.15	135.514	33.93166667	-1.997737705	Down	0.876289593	CTSC
144811	4180.09	0.838	12.535	3.902867942	Up	0.875593456	LACC1
727936	1547	0.45	10.52666667	4.547979859	Up	0.874187261	GXYLT2
27345	4725	15.64	1.601666667	-3.287594677	Down	0.874157095	KCNMB4
390	2742.83	341.064	86.39833333	-1.980967096	Down	0.873913447	RND3
4069	1516	23.186	3.566666667	-2.700605655	Down	0.873850795	LYZ
5118	1651	485.084	126.31	-1.941265732	Down	0.872864601	PCOLCE
9315	2028.92	24.354	98.08833333	2.009922806	Up	0.87285764	NREP
10468	1855.79	1.262	13.85166667	3.45627576	Up	0.872850679	FST

10687	4846	2.452	18.88	2.944827881	Up	0.872811231	PNMA2
26355	838	13.346	59.67	2.160598368	Up	0.872741617	FAM162A
4330	7569	0.1	8.836666667	6.465430359	Up	0.872238079	MN1
7980	2444	6.088	31.84333333	2.3869511	Up	0.871697413	TFPI2
8519	733	491.498	129.5066667	-1.92415918	Down	0.871521058	IFITM1
4222	2157	10.67	0.531666667	-4.326894348	Down	0.871423599	MEOX1
94122	4709	16.55	1.92	-3.107653001	Down	0.871337742	SYTL5
4609	2379	15.56	66.26333333	2.090368719	Up	0.871179951	MYC
4188	1662.48	0.754	11.62166667	3.946108647	Up	0.87091774	MDFI
3672	4811	57.602	13.13666667	-2.132519656	Down	0.870335306	ITGA1
5947	881.35	195.99	49.86333333	-1.974728811	Down	0.870161272	RBP1
1690	2558	12.24	0.915	-3.741688004	Down	0.869984917	COCH
2171	751	60.06	13.94833333	-2.106311728	Down	0.869977956	FABP5
3776	3274	1.484	14.74333333	3.312499744	Up	0.869831767	KCNK2
130497	1911	1.002	12.59666667	3.652087604	Up	0.869564915	OSR1
25891	3065.85	221.738	57.94	-1.936224441	Down	0.869325908	PAMR1
54331	3903	1.062	12.65833333	3.575231793	Up	0.868975519	GNG2
9120	3828	9.144	0.236666667	-5.271896975	Down	0.868706346	SLC16A6
55816	1766.03	0.146	8.755	5.906066904	Up	0.868615849	DOK5
1462	9549.9	39.244	154.065	1.972995149	Up	0.868179603	VCAN
7345	1141	82.858	306.2133333	1.8858242	Up	0.867850099	UCHL1
126868	3229	15.518	1.753333333	-3.145770427	Down	0.867321035	MAB21L3
6578	4234	38.624	7.95	-2.280470816	Down	0.867170205	SLCO2A1
7474	6151.31	176.57	46.21	-1.933963249	Down	0.867061144	WNT5A
9962	6953	3.22	20.74833333	2.687862859	Up	0.866907994	SLC23A2
1E+08	1221	26.25	4.743333333	-2.468344262	Down	0.866501914	LIMS4
2200	11695	33.65	129.115	1.939978206	Up	0.864842789	FBN1
1829	5885	0.946	11.875	3.64994352	Up	0.864411185	DSG2
341676	2963	28.766	5.775	-2.316471772	Down	0.86433693	NEK5
23240	5034	0.586	10.34166667	4.141424235	Up	0.864035271	KIAA0922
633	2470	118.78	418.8833333	1.818256545	Up	0.864007425	BGN
4257	945.12	49.854	183.0316667	1.876312106	Up	0.863961016	MGST1

9843	4302.62	2.142	16.485	2.944123502	Up	0.863489964	HEPH
586	9370.19	5.5	27.70166667	2.332469255	Up	0.863063	BCAT1
81606	2956	302.956	86.09166667	-1.815162776	Down	0.862666203	LBH
7263	1140.3	106.674	28.06333333	-1.9264503	Down	0.861993271	TST
3429	632.78	51.824	12.795	-2.018040226	Down	0.861364427	IFI27
10974	672	5.24	26.175	2.32055082	Up	0.860111382	ADIRF
3939	2225.19	312.362	1051.426667	1.751057371	Up	0.859937348	LDHA
3911	11445	38.798	8.91	-2.122484948	Down	0.858709827	LAMA5
4837	1579	43.362	153.8533333	1.827052496	Up	0.858691263	NNMT
3398	1402	19.436	75.43833333	1.956566465	Up	0.8585706	ID2
3655	5815.7	36.062	8.058333333	-2.161926022	Down	0.858301427	ITGA6
1535	778	360.054	106.1166667	-1.762562031	Down	0.858299107	CYBA
347	1148	11.552	0.99	-3.544570312	Down	0.858250377	APOD
1291	4246	172.69	578.5483333	1.744252948	Up	0.858185404	COL6A1
27147	3770.87	40.892	9.665	-2.080976988	Down	0.857930154	DENND2A
115207	6255	1.53	13.70833333	3.16344962	Up	0.857572804	KCTD12
1903	4404	102.288	28.05333333	-1.866392795	Down	0.857503191	S1PR3
154141	4334	12.006	1.113333333	-3.430798066	Down	0.857412693	MBOAT1
9052	2856	137.762	38.975	-1.821557065	Down	0.856964845	GPRC5A
5817	5567.32	142.132	40.61	-1.807324471	Down	0.856674788	PVR
5152	1590	12.726	1.32	-3.269169192	Down	0.856672468	PDE9A
2192	2680.88	178.5	51.7	-1.787687889	Down	0.856584291	FBLN1
650	3191	21.762	3.926666667	-2.470434113	Down	0.856298875	BMP2
9022	813	85.302	22.97166667	-1.892724035	Down	0.85612484	CLIC3
2009	4479	20.32	3.416666667	-2.572238993	Down	0.856038984	EML1
10135	4593	66.4	17.265	-1.943332907	Down	0.855243068	NAMPT
8644	1251	0.838	10.675	3.671142016	Up	0.854546931	AKR1C3
745	5816.29	6.986	31.61666667	2.178146725	Up	0.854419306	MYRF
5764	1759.47	40.058	9.801666667	-2.030991403	Down	0.854347372	PTN
9627	2867.64	12.368	1.278333333	-3.274276242	Down	0.854157095	SNCAIP
79901	4203.75	15.362	59.60333333	1.956026961	Up	0.853871679	CYBRD1
65010	2567.45	20.008	3.453333333	-2.53451546	Down	0.853686042	SLC26A6

51129	1869.01	4.084	21.735	2.411965324	Up		0.85341919	ANGPTL4
1306	5422	1.126	11.61	3.36608924	Up		0.853047917	COL15A1
51400	2744	179.946	53.73	-1.74376429	Down		0.852683606	PPME1
2354	3742.86	142.556	42.52166667	-1.745258711	Down		0.851391113	FOSB
9334	4743	9.104	37.04666667	2.024771277	Up		0.85122636	B4GALT5
10512	5189	3.612	19.99666667	2.468889733	Up		0.851008238	SEMA3C
5460	2075	12.734	1.438333333	-3.146215706	Down		0.850850447	POU5F1
8532	2306.14	95.574	27.36666667	-1.804198479	Down		0.850476853	CPZ
81619	5507.61	51.72	13.33833333	-1.955143867	Down		0.850402599	TSPAN14
6706	650	0.01	7.065	9.46454575	Up		0.850168233	SPRR2G
10516	2637	288.224	89.74333333	-1.683313796	Down		0.85010326	FBLN5
348	1234	12.498	1.408333333	-3.149636499	Down		0.849288781	APOE
2006	3726.09	7.762	32.635	2.071919701	Up		0.849260935	ELN
4771	5986	59.794	16.28666667	-1.876309362	Down		0.848780601	NF2
2745	1121.17	448.4	143.5866667	-1.64286449	Down		0.848453417	GLRX
27065	2453.94	0.302	8.085	4.742627319	Up		0.848353637	NSG1
2023	2206.56	306.132	938.0866667	1.615567357	Up		0.848098387	ENO1
2152	2383.61	74.958	237.7283333	1.665159494	Up		0.848086785	F3
3142	2308	24.27	4.976666667	-2.285922444	Down		0.847576285	HLX
715	2526	68.406	217.2266667	1.667006442	Up		0.847534517	C1R
84081	2526	33.996	8.435	-2.010905034	Down		0.847392969	NSRP1
1E+08	1155	117.218	35.34833333	-1.729480028	Down		0.847186449	LOC101928120
23414	4507	1.668	13.215	2.985985231	Up		0.846754844	ZFPM2
9249	1572.27	54.23	173.5633333	1.678299123	Up		0.846752523	DHRS3
6196	5667.27	7.774	31.95833333	2.039463159	Up		0.84658313	RPS6KA2
152	1958	23.456	4.766666667	-2.298904362	Down		0.846193294	ADRA2C
5214	2492.93	53.72	170.7633333	1.66846702	Up		0.845689755	PFKP
4504	599	0.01	6.79	9.407267764	Up		0.845158371	MT3
79365	3796	1.274	11.49333333	3.173360091	Up		0.845070194	BHLHE41
3570	3978.27	13.174	1.755	-2.90815052	Down		0.844408864	IL6R
283991	1604	50.544	13.805	-1.872348954	Down		0.844097923	UBALD2
9945	3115	4.718	22.94166667	2.28172288	Up		0.844060796	GFPT2

10669	1668.78	1.534	12.25833333	2.998392453	Up	0.843868198	CGREF1
8140	4575	15.926	56.69333333	1.831795133	Up	0.843745214	SLC7A5
4035	14905	43.03	137.15	1.672339877	Up	0.843684882	LRP1
3400	3891	5.014	23.56666667	2.232713813	Up	0.842930734	ID4
114801	2988	10.846	41.47666667	1.935136879	Up	0.842919132	TMEM200A
8840	5194	1.324	11.68	3.141065247	Up	0.842810071	WISP1
9360	2717	37.804	9.538333333	-1.986729786	Down	0.84249913	PPIG
2744	4601.6	17.38	60.725	1.804862503	Up	0.842459682	GLS
143241	787	11.54	1.288333333	-3.163065405	Down	0.842109293	DYDC1
3620	1944	8.798	0.573333333	-3.939729537	Down	0.842086089	IDO1
56241	3201	18.904	3.746666667	-2.335012	Down	0.841568627	SUSD2
55214	3351.33	70.492	21.04833333	-1.743753537	Down	0.84106973	P3H2
4601	3393.09	4.882	22.77833333	2.22211799	Up	0.840607959	MXI1
5730	837	71.086	21.35833333	-1.734766384	Down	0.839860773	PTGDS
64770	4238.69	41.956	11.34666667	-1.886608605	Down	0.839812043	CCDC14
7678	2243.49	20.85	4.205	-2.309869678	Down	0.839709943	ZNF124
669	1800	47.246	13.205	-1.83910789	Down	0.839531268	BPGM
4286	4551.59	16.932	3.08	-2.458750138	Down	0.839215686	MITF
54532	6612	56.054	16.315	-1.78061834	Down	0.839162316	USP53
3814	731	25.084	5.806666667	-2.110985301	Down	0.839099664	KISS1
54978	3912	53.508	15.42166667	-1.794795915	Down	0.838735352	SLC35F6
3569	1192.27	23.47	76.44	1.703509936	Up	0.83872607	IL6
147968	3129	91.118	28.565	-1.673487551	Down	0.838647175	CAPN12
80210	2929.27	98.878	31.5	-1.650297734	Down	0.838039216	ARMC9
401089	4075	46.484	13.17166667	-1.819296312	Down	0.837746838	FOXL2NB
22801	5035	44.258	134.595	1.604614654	Up	0.837298991	ITGA11
79944	6111	24.178	5.601666667	-2.109766868	Down	0.837117995	L2HGDH
57088	3358.87	1.258	11.01833333	3.130702187	Up	0.837013575	PLSCR4
3554	4868.8	12.756	45.03833333	1.819977438	Up	0.836749043	IL1R1
6741	1735.29	94.626	30.32666667	-1.641649708	Down	0.836561086	SSB
4744	3721	0.458	8.028333333	4.131681015	Up	0.836161968	NEFH
9524	1181.5	119.334	39.25333333	-1.604118071	Down	0.836043625	TECR

125965	1679	8.944	0.693333333	-3.689299161	Down	0.836004177	COX6B2
8862	3238	0.052	6.455	6.955761662	Up	0.835605059	APLN
4071	1712	18.344	60.33833333	1.717766587	Up	0.835598097	TM4SF1
10982	4220.89	6.38	25.92666667	2.022808405	Up	0.835570252	MAPRE2
200315	1390	12.32	1.753333333	-2.812830052	Down	0.835479754	APOBEC3A
84935	2394	13.778	47.51166667	1.785915333	Up	0.835468152	MEDAG
1E+08	1360	23.824	5.6	-2.088916927	Down	0.83530804	UPK3BL
3426	2218.31	10.376	37.87333333	1.867932018	Up	0.835182736	CFI
51776	6698.56	72.524	22.735	-1.673543494	Down	0.834985497	ZAK
57561	4294	4.222	20.11666667	2.252392786	Up	0.834493561	ARRDC3
4340	1942.08	10.792	1.248333333	-3.11188713	Down	0.834393781	MOG
140885	3892.94	14	47.61666667	1.766039803	Up	0.833892563	SIRPA
199731	2176	39.958	11.205	-1.834341726	Down	0.833683722	CADM4
729993	6698.07	24.982	6.153333333	-2.021448928	Down	0.833602506	SHISA9
6711	10077.66	18.122	58.71833333	1.696068835	Up	0.833249797	SPTBN1
57157	5017.72	57.092	17.51166667	-1.704972204	Down	0.832627915	PHTF2
11221	2680	13.622	2.256666667	-2.593673299	Down	0.832326256	DUSP10
11185	2606.61	16.254	3.131666667	-2.375792233	Down	0.83219399	INMT
728888	3497	89.376	29.42333333	-1.602926782	Down	0.83214294	NPIPB11
136	1885	4.326	20.015	2.209976045	Up	0.831961945	ADORA2B
29126	3610.51	16.074	3.068333333	-2.389201861	Down	0.83193642	CD274
165631	4180	31.798	8.375	-1.924774932	Down	0.831537301	PARP15
9636	685	42.54	12.37	-1.781974533	Down	0.831395754	ISG15
85300	5049	10.598	1.275	-3.05522288	Down	0.831323819	ATCAY
51171	1277	11.756	1.68	-2.806864126	Down	0.830975751	HSD17B14
91574	1669.5	30.798	8.1	-1.926842853	Down	0.830804038	C12orf65
7078	5496	110.854	324.72	1.550535417	Up	0.830681053	TIMP3
1E+08	1948	6.102	0.01	-9.25313837	Down	0.830630003	TNFAIP8L2-SCNM1
353322	953	2.5	14.52166667	2.538207042	Up	0.830502378	ANKRD37
1346	783	35.478	9.878333333	-1.844585126	Down	0.830117183	COX7A1
55365	1047	0.884	9.22	3.382648476	Up	0.829787678	TMEM176A
28959	1323.85	0.832	9.115	3.453587223	Up	0.829778397	TMEM176B

10752	7430.36	0.01	6.061666667	9.24357071	Up	0.829618285	CHL1
7145	10107.03	27.546	82.105	1.575627244	Up	0.829523147	TNS1
51575	3205	14.832	2.685	-2.465719156	Down	0.829165796	ESF1
1012	4016.61	21.922	67.51	1.622721794	Up	0.828989442	CDH13
116039	1877	17.864	3.635	-2.297027887	Down	0.828905906	OSR2
8771	1155	44.814	13.465	-1.734735274	Down	0.828402367	TNFRSF6B
379	1602	4.98	21.18666667	2.088938977	Up	0.828286344	ARL4D
55366	5240	0.88	9.065	3.364731591	Up	0.827388328	LGR4
54491	7064	9.448	0.985	-3.261813335	Down	0.827019376	FAM105A
716	2813.68	50.404	149.225	1.56587912	Up	0.827012414	C1S
23743	2459	20.65	4.855	-2.088598581	Down	0.826710755	BHMT2
3778	5468.14	2.686	14.615	2.443918619	Up	0.826552964	KCNMA1
5179	1365	13.752	2.343333333	-2.553007356	Down	0.826371969	PENK
63874	2543	10.722	36.70166667	1.775271539	Up	0.826244344	ABHD4
57568	6612	0.646	8.211666667	3.668068996	Up	0.826112078	SIPA1L2
4059	2572.73	59.178	19.21666667	-1.622702832	Down	0.826030862	BCAM
152330	5223	9.302	0.955	-3.283968302	Down	0.825896276	CNTN4
1759	3865.05	19.582	4.493333333	-2.123670126	Down	0.825285996	DNM1
3936	3808	25.998	6.65	-1.966974397	Down	0.825230305	LCP1
4853	11474	13.376	43.555	1.703191593	Up	0.825065553	NOTCH2
23643	621.27	2.316	13.53833333	2.547342985	Up	0.824872955	LY96
1917	2022	3.04	15.22	2.32382513	Up	0.824631628	EEF1A2
8379	2658.69	27.246	7.185	-1.922984381	Down	0.824483119	MAD1L1
4237	1124	62.968	21.03333333	-1.58194134	Down	0.824415826	MFAP2
150	3889	21.274	5.246666667	-2.019618278	Down	0.824206985	ADRA2A
10235	2262.13	9.664	1.081666667	-3.159364477	Down	0.824000464	RASGRP2
7262	937	50.524	16.24666667	-1.636825112	Down	0.823768419	PHLDA2
4061	1192.86	613.11	221.715	-1.467439557	Down	0.823733612	LY6E
139728	1551.68	1.026	9.311666667	3.182008684	Up	0.823694164	PNCK
442213	3140.78	9.892	1.231666667	-3.005650376	Down	0.822726534	PTCHD4
9669	4698	46.212	14.73666667	-1.648857295	Down	0.822125537	EIF5B
171019	5234	6.21	0.151666667	-5.355617319	Down	0.822048962	ADAMTS19

5064	2847.62	11.944	38.99833333	1.707126398	Up	0.822044321	PALM
2256	2652.41	1.626	11.21833333	2.786459193	Up	0.821728739	FGF11
10397	3123	44.082	127.6316667	1.533724735	Up	0.821712496	NDRG1
26993	2208.01	41.672	12.97333333	-1.683529133	Down	0.821670727	AKAP8L
10848	3120.07	11.718	38.44833333	1.714194706	Up	0.82163128	PPP1R13L
6518	2454	0.104	6.028333333	5.857103759	Up	0.821543102	SLC2A5
8470	4731.81	0.5	7.47	3.901108243	Up	0.820888734	SORBS2
10221	3214.94	35.938	10.85333333	-1.727371924	Down	0.820768071	TRIB1
3371	8605	28.906	85.565	1.565651806	Up	0.820148509	TNC
9976	2052	26.51	7.223333333	-1.875800017	Down	0.820076575	CLEC2B
389177	1902	47.198	15.30333333	-1.624879796	Down	0.82005337	TMEM212
54737	4281	27.742	7.716666667	-1.846022107	Down	0.820034807	MPHOSPH8
3206	2648	18.462	4.296666667	-2.103269087	Down	0.819860773	HOXA10
57559	2094	34.962	10.525	-1.731967483	Down	0.819767955	STAMBPL1
5763	1224	838.634	311.8966667	-1.426973187	Down	0.819262095	PTMS
219623	5162	7.366	0.498333333	-3.885698414	Down	0.819027729	TMEM26
50863	3151.77	0.906	8.77	3.274993887	Up	0.818872259	NTM
6038	1831.14	6.756	25.19333333	1.898800785	Up	0.818784082	RNASE4
83982	469	118.778	41.74	-1.50876514	Down	0.818702866	IFI27L2
2318	9147.13	47.222	133.27	1.496821006	Up	0.818688943	FLNC
23710	1885	145.99	51.95166667	-1.490627613	Down	0.818445295	GABARAPL1
1303	9612.43	533.576	198.2683333	-1.428239502	Down	0.818396566	COL12A1
3134	1236.15	48.51	16.15	-1.586748015	Down	0.818236454	HLA-F
7089	2514.48	13.668	2.62	-2.383163436	Down	0.818190045	TLE2
445329	1397	13.884	2.723333333	-2.349977788	Down	0.817995127	SULT1A4
126433	2376	13.726	2.653333333	-2.371033425	Down	0.817985845	FBXO27
153769	3043	0.574	7.493333333	3.706484988	Up	0.817303632	SH3RF2
58498	619	0.366	6.798333333	4.215265548	Up	0.817083188	MYL7
115123	4196	0.292	6.488333333	4.473807665	Up	0.816568047	3-Mar
83604	4068	15.234	45.72833333	1.585793537	Up	0.81642882	TMEM47
90199	1269	9.234	1.116666667	-3.047757138	Down	0.816398654	WFDC8
8613	3324	2.534	13.28666667	2.39049078	Up	0.816194454	PLPP3

58517	4303	45.426	15.23333333	-1.576286609	Down	0.815639865	RBM25
3394	2678	0.126	5.873333333	5.54268388	Up	0.815491356	IRF8
4728	824	110.612	39.38	-1.489972892	Down	0.815454229	NDUFS8
55690	4511	39.376	110.4316667	1.487765461	Up	0.81541014	PACS1
57608	9300	7.462	26.36	1.820716105	Up	0.815150249	KIAA1462
3728	3284.38	89.482	31.41666667	-1.510067385	Down	0.814841629	JUP
9630	2492	11.29	1.888333333	-2.579860126	Down	0.814700081	GNA14
387763	1361	31.47	9.533333333	-1.722924532	Down	0.814669915	C11orf96
7046	6479.49	6.89	24.87666667	1.852217297	Up	0.81438914	TGFBR1
9580	4088	13.458	2.698333333	-2.318323541	Down	0.814191902	SOX13
627	4081.85	6.45	23.725	1.879037022	Up	0.813992342	BDNF
8321	4350	2.684	13.36833333	2.316363035	Up	0.813586263	FZD1
1000	4018.97	61.912	168.0133333	1.440284758	Up	0.813579302	CDH2
3437	2503.45	31.698	9.79	-1.695011051	Down	0.813147697	IFIT3
3575	4617	16.766	3.876666667	-2.112649938	Down	0.813001508	IL7R
23768	8085	1.486	10.27666667	2.789866368	Up	0.81280659	FLRT2
55568	5966	10.304	32.95	1.677073967	Up	0.812672004	GALNT10
5670	1612	8.912	1.065	-3.064895802	Down	0.812637197	PSG2
3306	2802	68.892	23.89333333	-1.527728325	Down	0.812428356	HSPA2
1E+08	1739	7.16	0.525	-3.76957026	Down	0.812198631	MTRNR2L5
8605	2519.83	12.086	2.15	-2.490928282	Down	0.812026917	PLA2G4C
10100	3214.18	0.154	5.84	5.244966113	Up	0.811959624	TSPAN2
57106	1368	35.946	11.61333333	-1.630049116	Down	0.811727579	NAT14
283869	1033	6.976	0.48	-3.861293729	Down	0.811611556	NPW
84969	2490.67	2.084	11.90333333	2.51393845	Up	0.811576749	TOX2
60494	2518	12.826	2.498333333	-2.36003343	Down	0.811381831	CCDC81
79625	2911	0.182	5.903333333	5.019519451	Up	0.811145144	NDNF
1839	2381	17.606	4.341666667	-2.019746301	Down	0.811066249	HBEGF
9464	2368	24.714	7.033333333	-1.813048034	Down	0.810980392	HAND2
9242	2075	3.844	16.43166667	2.095798579	Up	0.81096879	MSC
3880	1490	667.59	258.6183333	-1.368137794	Down	0.810632324	KRT19
64359	2831.89	78.572	27.90666667	-1.493405473	Down	0.810437406	NXN

51310	2244.65	38.118	12.73666667	-1.581484668	Down	0.809787678	SLC22A17
90226	1546	7.488	0.671666667	-3.478763097	Down	0.809720385	UCN2
51655	1740	9.118	1.156666667	-2.978744247	Down	0.809660053	RASD1
112817	2012	8.764	1.081666667	-3.018333507	Down	0.809620606	HOGA1
10630	2825.08	81.944	29.44666667	-1.476533986	Down	0.809571876	PDPN
11145	1324.11	2.904	14.13166667	2.282818266	Up	0.809367676	PLA2G16
10659	8029.42	0.114	5.598333333	5.61789166	Up	0.809205244	CELF2
341116	2296	9.192	1.211666667	-2.923385935	Down	0.808861817	MS4A10
24147	2701	30.624	9.645	-1.666809592	Down	0.808687783	FJX1
64409	3518	6.25	0.31	-4.333516069	Down	0.808372201	WBSCR17
284656	2082	6.564	0.405	-4.018581426	Down	0.80800789	EPHA10
513	877.61	137.458	51.89333333	-1.405369759	Down	0.807272305	ATP5D
58489	2408	29.606	9.316666667	-1.668003802	Down	0.807075067	ABHD17C
344148	7611	11.17	1.916666667	-2.542957825	Down	0.806699153	NCKAP5
4908	1349	3.044	14.205	2.222358568	Up	0.806622578	NTF3
55686	3213	11.046	1.873333333	-2.559844498	Down	0.806365008	MREG
440498	766	34.348	11.37833333	-1.593936846	Down	0.806286112	HSBP1L1
57414	1826.37	51.722	17.96666667	-1.52545529	Down	0.805529644	RHBDD2
114960	2058	7.228	0.636666667	-3.504986369	Down	0.805513401	TSGA13
3221	1689	0.26	5.958333333	4.518325308	Up	0.805466992	HOXC4
5046	4532	0.388	6.423333333	4.049193608	Up	0.805420582	PCSK6
165	4102	304.804	119.8066667	-1.347173646	Down	0.80530456	AEBP1
1E+08	2130	61.334	21.87833333	-1.487184202	Down	0.805214062	BCL2L2-PABPN1
84251	10174	7.758	0.795	-3.28665801	Down	0.804965773	SGIP1
54873	2601	19.548	5.176666667	-1.916925681	Down	0.80469428	PALMD
3931	1354	15.64	3.76	-2.056435946	Down	0.804281239	LCAT
116372	3624.41	13.728	2.92	-2.233081184	Down	0.804223228	LYPD1
7225	4612	16.304	4.086666667	-1.996229478	Down	0.804158255	TRPC6
127435	3186.39	1.292	9.061666667	2.810170352	Up	0.803747535	PODN
3091	4081.16	88.116	224.805	1.351198213	Up	0.803557257	HIF1A
10518	1624.93	18.076	4.58	-1.980655959	Down	0.803137255	CIB2
949	2747.67	78.896	29.27833333	-1.430118727	Down	0.802970182	SCARB1

2597	1421.19	2197.64	5432.58	1.30568244	Up	0.802865762	GAPDH
598	2606.27	84.36	31.57333333	-1.417852515	Down	0.802828634	BCL2L1
7431	2151	1286.506	3197.278333	1.313386155	Up	0.802777584	VIM
9295	2808.19	78.014	28.97666667	-1.428841402	Down	0.802759021	SRSF11
51574	2216.69	28.158	8.98	-1.648757516	Down	0.802642998	LARP7
23136	4000.08	0.602	7.003333333	3.540206364	Up	0.802125537	EPB41L3
6575	3672.77	51.59	18.19166667	-1.503813722	Down	0.802106973	SLC20A2
2879	939.03	262.162	104.3616667	-1.328866695	Down	0.80178675	GPX4
29886	4756	18.184	4.711666667	-1.948360207	Down	0.801700893	SNX8
388325	2206.85	12.746	2.563333333	-2.313951563	Down	0.801668407	SCIMP
3897	5059.24	30.086	9.9	-1.60359188	Down	0.801417798	L1CAM
253982	1562	19.164	5.19	-1.884592275	Down	0.800496577	ASPHD1
9147	4803	21.75	6.338333333	-1.778839963	Down	0.800199559	NEMF
10577	921	396.872	160.0866667	-1.309820628	Down	0.800106741	NPC2
5368	1022	0.01	4.958333333	8.953711452	Up	0.800041768	PNOC
283987	3369	7.228	0.706666667	-3.354494742	Down	0.800037127	HID1

Table 3 The well-chosen top 8 pathway enrichment of DEGs between EnSC-Control and EnSC-EM-EC

No	Pathway	DEGs genes with pathway annotation (473)	P value	Q value	The specific DEGs involved in pathways (Gene ID_Gene symbol)
**1**	**Complement and coagulation cascades**	15 (3.17%)	2.620016e-06	0.000662864	3075_CFH, 629_CFB, 5054_SERPINE1, 716_C1S, 5328_PLAU, 90199_WFDC8, 2159_F10, 7980_TFPI2, 2151_F2RL2, 81606_LBH, 718_C3, 2152_F3, 715_C1R, 5648_MASP1, 3426_CFI
**2**	**MicroRNAs in cancer**	19 (4.02%)	2.133831e-05	0.002699296	1545_CYP1B1, 3162_HMOX1, 4853_NOTCH2, 54541_DDIT4, 3371_TNC, 4609_MYC, 7078_TIMP3, 7148_TNXB, 101928120_LOC101928120, 57493_HEG1, 5328_PLAU, 7431_VIM, 23414_ZFPM2, 400916_CHCHD10, 2744_GLS, 9358_ITGBL1, 57451_TENM2, 5155_PDGFB, 3236_HOXD10
**3**	**ECM-receptor interaction**	14 (2.96%)	6.356155e-05	0.004108390	1311_COMP, 1291_COL6A1, 3371_TNC, 7148_TNXB, 3655_ITGA6, 7058_THBS2, 2335_FN1, 3672_ITGA1, 9358_ITGBL1, 22801_ITGA11, 3908_LAMA2, 1690_COCH, 57451_TENM2, 3911_LAMA5
**4**	**Glycolysis / Gluconeogenesis**	10 (2.11%)	6.495479e-05	0.004108390	2597_GAPDH, 2023_ENO1, 669_BPGM, 3939_LDHA, 2026_ENO2, 5230_PGK1, 5214_PFKP, 219_ALDH1B1, 220_ALDH1A3, 5236_PGM1
**5**	**HIF-1 signaling pathway**	13 (2.75%)	0.0001239916	0.006273975	3162_HMOX1, 2597_GAPDH, 2023_ENO1, 3939_LDHA, 1906_EDN1, 2026_ENO2, 5230_PGK1, 5054_SERPINE1, 5214_PFKP, 7076_TIMP1, 3091_HIF1A, 3570_IL6R, 3569_IL6

No	Pathway	DEGs genes with pathway annotation (473)	P value	Q value	Differentially expressed genes
**6**	**PI3K-Akt signaling pathway**	24 (5.07%)	0.0008668201	0.031329355	1311_COMP, 2256_FGF11, 1291_COL6A1, 54541_DDIT4, 3575_IL7R, 3371_TNC, 4609_MYC, 7148_TNXB, 54331_GNG2, 3655_ITGA6, 7058_THBS2, 2335_FN1, 3672_ITGA1, 4803_NGF, 3570_IL6R, 3569_IL6, 9358_ITGBL1, 22801_ITGA11, 3908_LAMA2, 598_BCL2L1, 1690_COCH, 57451_TENM2, 3911_LAMA5, 5155_PDGFB
**7**	**Cytokine-cytokine receptor interaction**	17 (3.59%)	0.001409798	0.043070608	3554_IL1R1, 1439_CSF2RB, 6347_CCL2, 6352_CCL5, 6376_CX3CL1, 3575_IL7R, 8771_TNFRSF6B, 650_BMP2, 27242_TNFRSF21, 9244_CRLF1, 6387_CXCL12, 55504_TNFRSF19, 3570_IL6R, 3569_IL6, 6372_CXCL6, 7046_TGFBR1, 5155_PDGFB
**8**	**Focal adhesion**	19 (4.02%)	0.001532156	0.043070608	1311_COMP, 1291_COL6A1, 3371_TNC, 58498_MYL7, 7148_TNXB, 101928120_LOC101928120, 3655_ITGA6, 2318_FLNC, 11346_SYNPO, 7058_THBS2, 2335_FN1, 3672_ITGA1, 9358_ITGBL1, 22801_ITGA11, 3908_LAMA2, 1690_COCH, 57451_TENM2, 3911_LAMA5, 5155_PDGFB
